# Similar Binding Modes of cGMP Analogues Limit Selectivity in Modulating Retinal CNG Channels via the Cyclic Nucleotide-Binding Domain

**DOI:** 10.1021/acschemneuro.3c00665

**Published:** 2024-04-05

**Authors:** Palina Pliushcheuskaya, Sandeep Kesh, Emma Kaufmann, Sophie Wucherpfennig, Frank Schwede, Georg Künze, Vasilica Nache

**Affiliations:** † Institute for Drug Discovery, Medical Faculty, University of Leipzig, Leipzig 04103, Germany; ‡ Institute of Physiology II, University Hospital Jena, Friedrich Schiller University Jena, Jena 07743, Germany; § BIOLOG Life Science Institute GmbH & Co KG, Bremen 28199, Germany; ∥ Interdisciplinary Center for Bioinformatics, University of Leipzig, Leipzig 04107, Germany; ⊥ Center for Scalable Data Analytics and Artificial Intelligence, University of Leipzig, Leipzig 04105, Germany

**Keywords:** retinal CNG channels, selective modulators, retinitis pigmentosa, cGMP analogues, patch-clamp technique, ligand docking

## Abstract

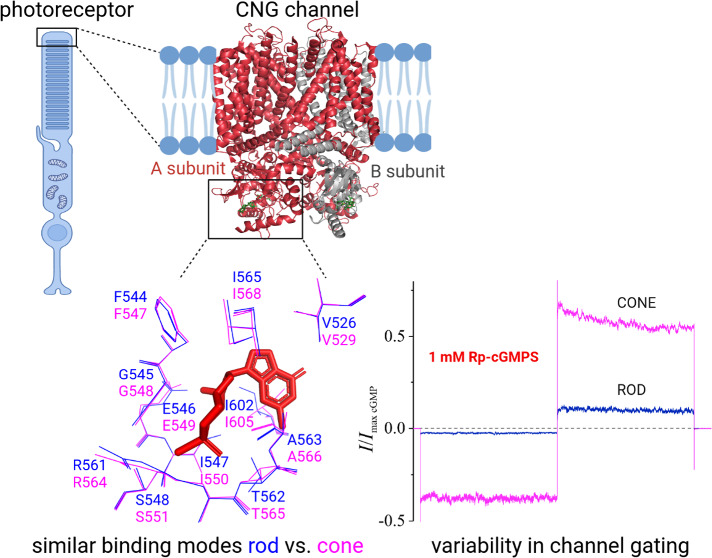

In treating *retinitis pigmentosa*, a genetic disorder causing progressive vision loss, selective inhibition of rod cyclic nucleotide-gated (CNG) channels holds promise. Blocking the increased Ca^2+^-influx in rod photoreceptors through CNG channels can potentially delay disease progression and improve the quality of life for patients. To find inhibitors for rod CNG channels, we investigated the impact of 16 cGMP analogues on both rod and cone CNG channels using the patch-clamp technique. Although modifications at the C8 position of the guanine ring did not change the ligand efficacy, modifications at the N1 and N^2^ positions rendered cGMP largely ineffective in activating retinal CNG channels. Notably, PET-cGMP displayed selective potential, favoring rod over cone, whereas Rp-cGMPS showed greater efficiency in activating cone over rod CNG channels. Ligand docking and molecular dynamics simulations on cyclic nucleotide-binding domains showed comparable binding energies and binding modes for cGMP and its analogues in both rod and cone CNG channels (CNGA1 vs CNGA3 subunits). Computational experiments on CNGB1a vs CNGB3 subunits showed similar binding modes albeit with fewer amino acid interactions with cGMP due to an inactivated conformation of their C-helix. In addition, no clear correlation could be observed between the computational scores and the CNG channel efficacy values, suggesting additional factors beyond binding strength determining ligand selectivity and potency. This study highlights the importance of looking beyond the cyclic nucleotide-binding domain and toward the gating mechanism when searching for selective modulators. Future efforts in developing selective modulators for CNG channels should prioritize targeting alternative channel domains.

## Introduction

Cyclic nucleotide-gated (CNG) channels are tetrameric nonselective cation channels that convert chemical signals, i.e., changes of intracellular cGMP or cAMP levels, into electrical signals that are passed on to the brain. The most studied CNG channels are the ones from photoreceptors and olfactory sensory neurons (OSNs), where they play an important role within the sensory transduction pathways.^1^ Retinal CNG channels are activated by cGMP, whereas in OSNs, the channels can be activated by both cAMP and cGMP.^2,3^ Based on their structural architecture, CNG channels belong to the family of cyclic nucleotide-binding domain (CNBD) channels. Although they differ considerably in their way of action, all members of this class, e.g., the CNG channels, the hyperpolarization-activated cyclic nucleotide-gated (HCN) channels, and the ether-à-go-go-type (KCNH) channels, possess a CNBD.^4^ The family of CNBD channels belong to the voltage-gated K^+^-channel superfamily.^5^ Despite containing the positively charged S4 domain, essential for detecting membrane voltage changes, CNG channels exhibit almost no voltage dependence.

**Figure 1:**
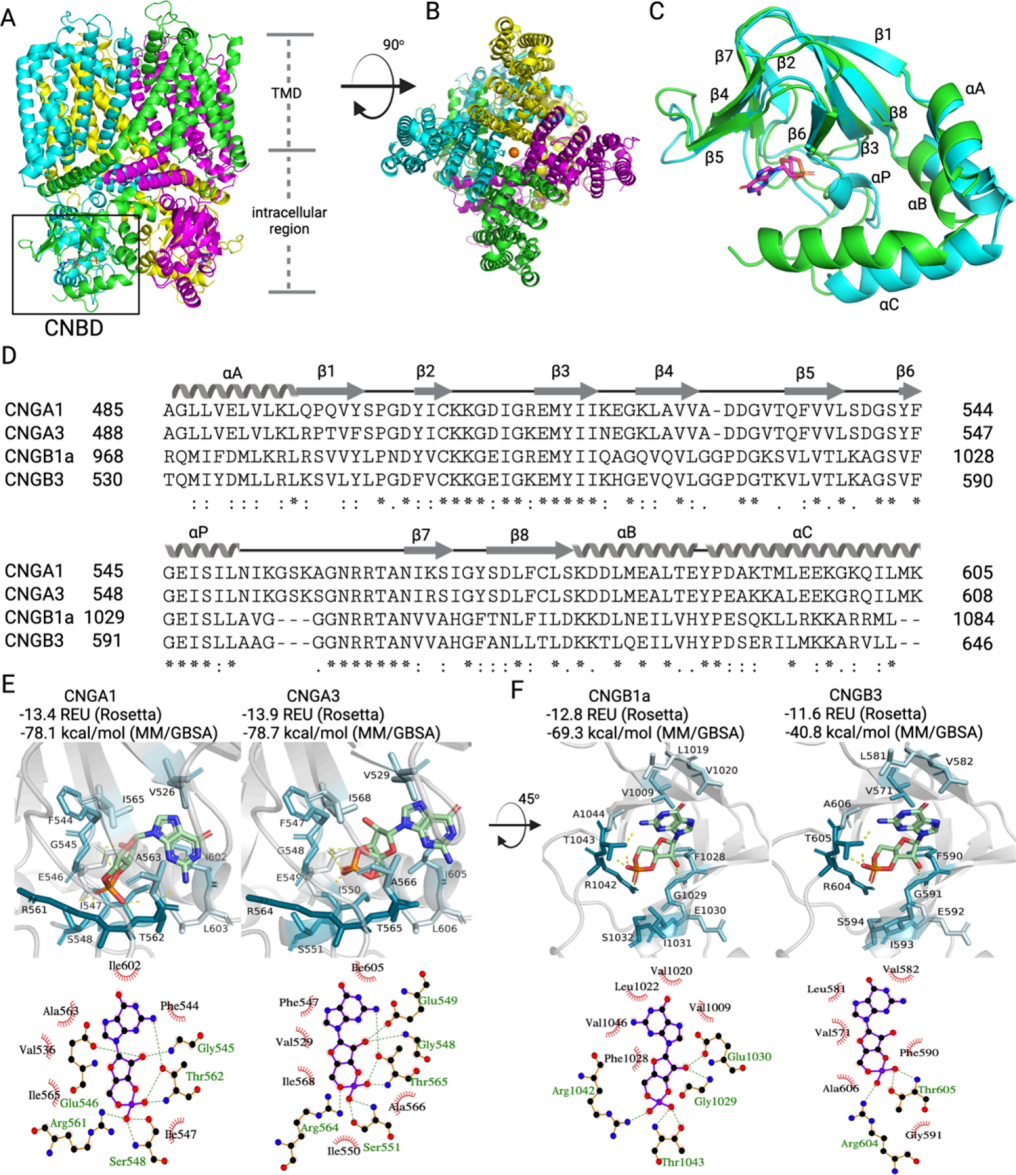
cGMP binding in heterotetrameric rod and cone CNG channels. (A) Overall structure of the CNG channel viewed from a direction parallel to the membrane plane (PDB: 7RHH(6)). (B) Overall structure of the CNG channel viewed from the extracellular side. (C) Zoom view of the cyclic nucleotide-binding domain (CNBD) in superimposed CNGA1 (green, PDB: 7RHH(6)) and CNGB1a (cyan, PDB: 7RHH(6)) subunits with cGMP bound. (D) Sequence alignment of the CNBDs of the CNGA1, CNGA3, CNGB1a, and CNGB3 subunits. (E, F) 3D (top) and 2D (bottom) diagrams of the cGMP binding mode in CNGA1/CNGA3 and CNGB1a/CNGB3 subunits, respectively. The Rosetta ligand docking score and the MM/GBSA binding energy of cGMP are listed above the plots. Residues that were found to be important for cGMP binding by MD MM/GBSA analysis are colored blue in the 3D diagrams, with darker colors representing more favorable energy values. Hydrophobic contacts are represented as red arcs and hydrogen bonds are depicted as green dashed lines between amino acid residues and the ligand in the 2D diagrams.

Rod and cone CNG channels share a similar core architecture. Each subunit is composed of six transmembrane segments, a pore domain, an intracellular C-linker domain, and an intracellular CNBD.^4^ The CNBD contains helices A, P, B, and C and a β-roll between helices A and B^6^ (Figure 1A–C). The cyclic nucleotide binds within the β-roll and is secured by the upward movement of the C-helix, effectively sealing the binding pocket like a lid.^6,7^ Ligand binding triggers the movement of the C-linker/gating ring closer to the transmembrane segments. As a result, S4 and S5 move away from the pore, which then causes S6 to dilate and the channel pore to open. In cones, the CNG channel contains three CNGA3 and one CNGB3 subunits, whereas in rods, the channel has three CNGA1 and one CNGB1a subunits.^1^ Furthermore, there is structural evidence that the rod and cone CNG channels possess a coil-coiled region formed by the C-terminal helices in the CNGA1 or CNGA3 subunits, respectively, which are important for establishing the 3:1 channel subunit stoichiometry.^8^ Interestingly, the helix following the C-helix in rod CNGB1a (termed the D-helix) contains a calmodulin (CaM) binding site, and a density fitting the C-lobe of CaM has been observed in one cryo-EM map of the rod CNG channel. It has been suggested that CaM could connect CNGB1 with one of the CNGA1 subunits participating in the coiled-coil structure, providing a hint on the structural mechanism of CaM modulation of the rod CNG channel.^9^

CNG channels are involved in the pathophysiological mechanism of many forms of inherited retinal diseases and are thus promising drug targets.^10,11^ In case of *retinitis pigmentosa* (RP), a faulty signaling pathway in rods triggers first rod degeneration followed shortly after by cones death, leading to partial or complete blindness.^12,13^ So far, more than 300 mutations to genes encoding for different proteins with important roles within the visual transduction cascade have been identified as the leading cause of retinal degeneration diseases (Retina Information Network, https://web.sph.uth.edu/RetNet/). A pathological cGMP accumulation in rod photoreceptors is the hallmark of this condition, which triggers an increased Ca^2+^-influx due to an exacerbated CNG-channel activity and finally cell death.^10,14−16^

Developing drugs that target rod CNG channels is, however, a challenging task due to the following reasons: first, because several CNG-channel isoforms have been found also in other types of neurons and in nonexcitable cells besides the retina, e.g., in neurons of the medial vestibular nucleus, in astrocytes, and in hippocampal neural stem cells,^17^ and second, because there are other cGMP targets, beside the CNG channels, with complex cellular functions, e.g., protein kinase G (PKG), and structurally very similar cyclic nucleotide-binding domains.^18^ The aim of recent pharmacological studies was to identify selective modulators for the rod CNG-channel isoform, ideally with no cross-reactivity with other intracellular targets. It was previously shown that Rp-8-Br-PET-cGMPS, a cGMP analogue with inhibitory effects on CNG channels, could efficiently delay the progress of rod degeneration in RP mice models.^19^ Although this compound failed to selectively modulate rod CNG channels only, the cGMP-analogue mixture containing Rp-8-Br-PET-cGMPS and 8-pCPT-cGMP, a concentration-dependent cone selective potent CNG channel agonist, could efficiently inhibit the rod photoreceptors.^20^ Another candidate for a selective modulation of rod CNG channels was the Ca^2+^-channel blocker l-*cis*-diltiazem.^21,22^ Unfortunately, l-*cis*-diltiazem not only failed to delay retinal degeneration on RP mice models but also showed toxic effects in photoreceptors, accelerating disease development.^23^ So far, there are no known selective inhibitors for either rod or cone CNG channels. Notably, Rp-cGMPS demonstrated its ability to activate rod channels, yet it did not elicit activation in olfactory CNG channels.^24^

The process of understanding the pharmacology of CNG channels is only in its early phase and was limited by the lack of structural information regarding these channels. This changed dramatically over the last years, when a long overdue wealth of structural data became available for retinal CNG channels.^6,7,25−28^ This together with atomistic computer modeling can significantly increase the speed and the effectiveness of designing new molecules that are able to bind selectively to the protein of interest. Herein we aimed to increase our understanding of the ligand selectivity mechanism of retinal CNG channels by investigating the effects and molecular interactions of 16 cGMP analogues on retinal CNG channels using electrophysiological studies of heterologously expressed CNG channels coupled with molecular modeling. Our systematic analysis gives a concise description of the structural CNBD features of rod and cone CNG channels, which are relevant for ligand binding. Furthermore, our findings are expected to have significant implications for the development of novel therapeutic approaches for RP, as targeting channel gating rather than cGMP binding is likely to be the most promising strategy for selectively modulating rod CNG channels in this disease.

## Results

### Characterization of cGMP Binding in Rod and Cone CNG Channels

Molecular interactions between cGMP and retinal CNG-channel structures were examined by means of ligand docking calculations and molecular dynamics (MD) simulations using as a starting point the binding pose of cGMP in the syn-configuration in the respective cryo-EM channel structures.^6,27^ We characterized and compared the interactions occurring between cGMP and residues within the binding pockets of the CNGA1/CNGB1a and CNGA3/CNGB3 subunits of rod and cone CNG channels, respectively. Figure 1 depicts the binding mode of cGMP to the CNBD of rod and cone CNGA-type and CNGB-type subunits. Overall, cGMP exhibits a similar interaction pattern in both retinal CNG-channel isoforms (Figure 1E,F).

The amine group at position 2 in the guanine moiety of cGMP interacts with T562 (αP-β7 loop) in the rod CNGA1 subunit, which corresponds to T565 in cone CNGA3 (Figure 1E). The same threonine residue introduces an additional hydrogen bond to the phosphate group of cGMP. Hydrogen bonds between the 2′-hydroxyl group of the ribose ring in cGMP and G545 and E546 (αP helix) are observed in the rod CNGA1 subunit (G548, E549 in cone CNGA3 subunit). Furthermore, S548 (αP) and R561 (αP-β7 loop) in rod CNGA1 and S551 and R564 in cone CNGA3, respectively, establish hydrogen bonds with oxygen atoms in the phosphate group of cGMP. Other residues that take part in the interactions between the CNBD and cGMP are mostly involved in hydrophobic contacts. F544 (β6 strand) and I602 (αC helix) in rod CNGA1 (F547 and I605 in cone CNGA3) interact with the guanine moiety of cGMP. I547 (αP) and A563 (αP-β7 loop) in rod CNGA1 (I550, A566 in cone CNGA3) are in contact with the phosphate group through van der Waals interactions.

**Table 1:**
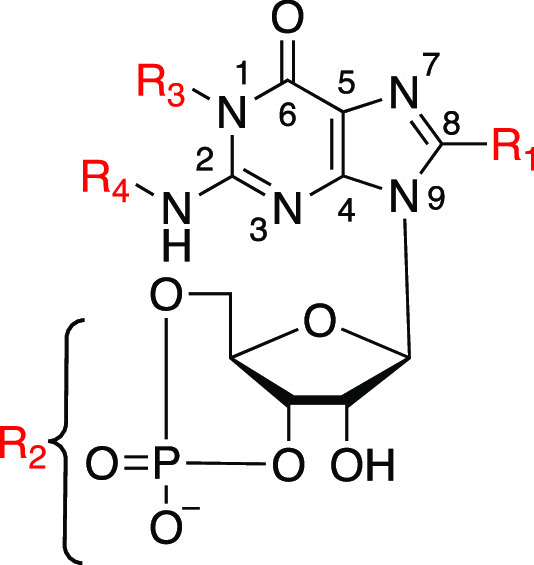

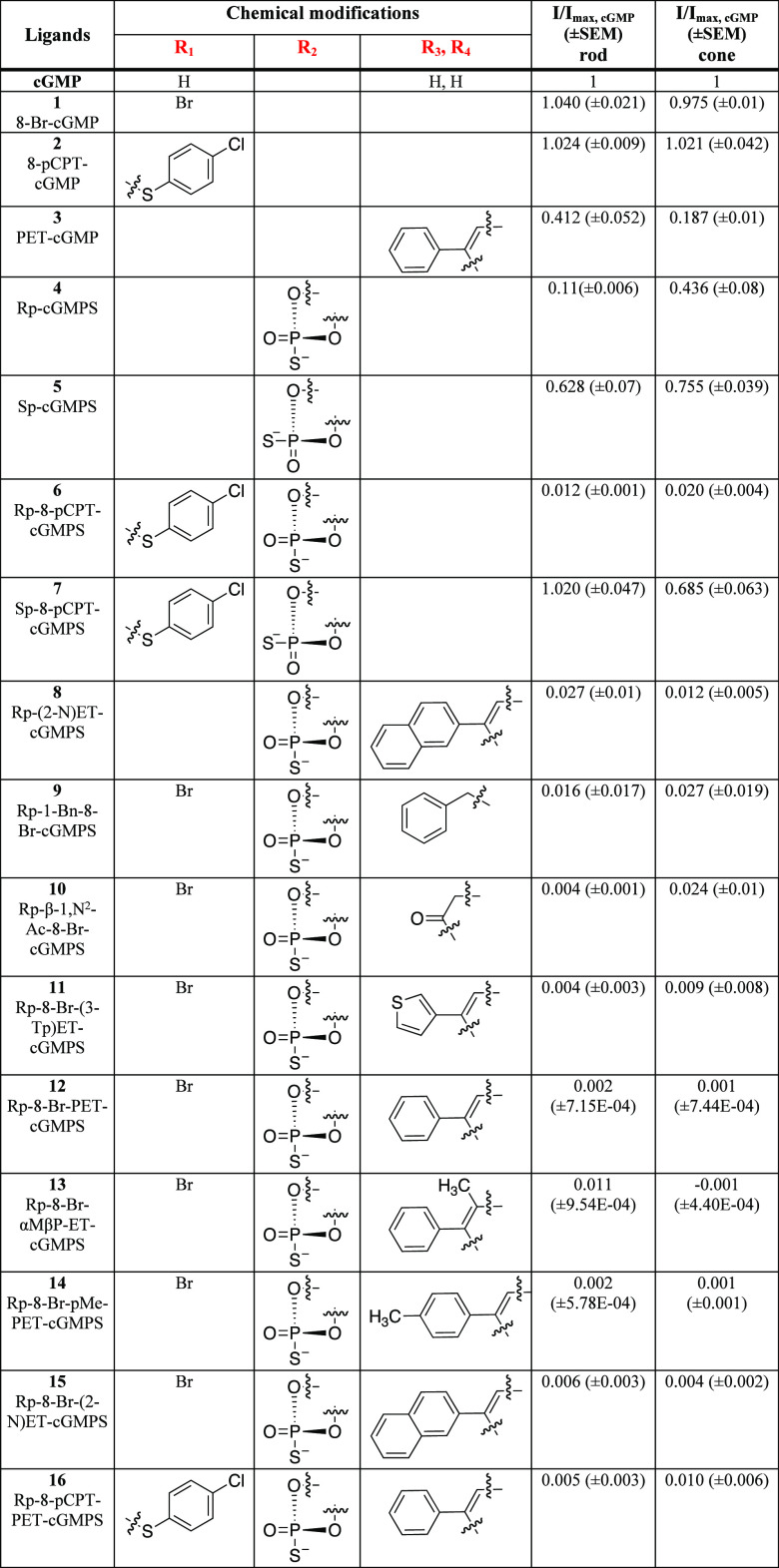
Chemical Structures and Corresponding Relative Currents in CNG Channels Elicited by the Guanosine-3′,5′-cyclic monophosphate (cGMP) and Its Analogues^a^

a R1–R4 represent chemical modifications present in the respective cGMP analogues.

We also investigated the interactions of cGMP in rod CNGB1a and cone CNGB3 subunits, respectively (Figure 1F). The same set of amino acid residues is involved in the binding of cGMP, e.g., T1043 (αP-β7 loop), G1029 (αP), and R1042 (αP-β7 loop) in CNGB1a and T605, G591, and R604 in CNGB3. However, there are less hydrophobic interactions with the residues in the C-helix; e.g., M1083 (CNGB1a) and L645 (CNGB3) that correspond to I602 in CNGA1 or I605 in CNGA3, respectively, fail to form contacts with the guanine moiety of cGMP. This is explained by the fact that computational simulations were conducted on the open I state structure of the CNG channels (PDB: 7RHH(6)), where CNGA subunits have a “C-helix up” (activated state) conformation, whereas the CNGB subunit remains in a “C-helix down” (inactivated state) conformation. In the open II state structure (PDB: 7RHI(6)), the CNGB1a subunit adopts a “C-helix up” conformation, more similar to that of the CNGA1 subunit, which allows the CNBD to form contacts with cGMP via its C-helix. We specifically chose to focus on the open I state structure of the CNGB1a subunit for several reasons. The C-helix is tilted by approximately 30° compared to that in the CNGA1 structure. This unique feature makes the open I state particularly intriguing for understanding the dynamics and interactions governing channel function in the absence of crucial contacts with the bound cGMP molecule. “C-helix down” and “C-helix up” conformational states have also been observed in the CNGB3 subunit of cone CNG channel.^28^ However, despite these intermediate states and missing densities, previous structural studies have consistently shown that the overall conformational changes within the CNBD of CNGB-type subunits do not result in significant pore opening, unlike their CNGA-type counterparts.^6,27^ Instead, these conformational changes tend to trigger only minor heterotetrameric channel activation.^29,30^ Interestingly, the loop connecting the β7 strand and P-helix binds to the C-helix in the CNGA1 and CNGA3 structures but not in the structures of the CNGB1a and CNGB3 subunits.^31^

Furthermore, in the design of our computational experiments, we omitted the coiled-coil and D-helix regions from the simulations. This decision was made due to the absence of a well-established mechanistic understanding regarding the interaction mode of CaM with the CNG channel. Additionally, the loops connecting those regions to the CNBD are not resolved in the available structures likely because of their high flexibility, thus compromising the accuracy of their modeling.

Visual analysis results of the interactions between cGMP and CNBDs of rod and cone CNG-channel isoforms were confirmed by performing per-residue decomposition analysis in Molecular Mechanics with Generalized Born and Surface Area Solvation (MM/GBSA) calculations (Figure 1E,F), which provided the contribution of each residue to the total binding free energy of cGMP. To this end, snapshots of the first 20 ns of the MD simulation were used, and separate configurations of protein, ligand, and protein–ligand complex were extracted to calculate the binding energy. According to the MM/GBSA computations, R561 (αP-β7 loop) in CNGA1 has the energy value with the highest magnitude (−12.0 kcal/mol) and therefore contributes the most to cGMP binding. The second, third, and fourth most important residues for cGMP binding are T562 (αP-β7 loop) (−9.2 kcal/mol), S548 (αP) (−8.0 kcal/mol), and I547 (αP) (−5.3 kcal/mol). They are followed by G545 (αP) (−3.4 kcal/mol), F544 (β6) (−2.7 kcal/mol), I602 (αC) (−2.4 kcal/mol), and A563 (αP- β7 loop) (−2.0 kcal/mol). The same pattern was observed for the CNBD of cone CNGA3, where the most important residues are R564 (αP-β7 loop), T565 (αP-β7 loop), S551 (αP), I550 (αP), G548 (αP), F547 (β6), I605 (αC), and A566 (αP-β7 loop), starting from the residue with the highest magnitude energy value in descending direction. The observation from MM/GBSA experiments that arginine at positions 561 and 564 (CNGA1, CNGA3) contributes most to the ligand binding agrees well with earlier studies by Tibbs et al.^32^ Tibbs and colleagues showed that arginine within the αP-β7 loop stabilizes ligand binding in a state-independent manner. This was confirmed by the substantial decrease in apparent ligand affinity upon mutating the arginine residue to neutral or oppositely charged amino acid residues. Results from the per-residue decomposition analysis were also confirmed by the estimation of the fraction of contacts between protein residues and cGMP that were observed over the simulation time within a cutoff distance of 3.5 Å. Heatmaps of the fraction of contacts are presented in the Supporting Information (Figure S1).

We also analyzed the MM/GBSA energies and fraction of contacts for the rod CNGB1a and cone CNGB3 subunits (Figures S2 and S3). Overall, the interaction patterns of cGMP in both CNG-channel isoforms of the CNGA and CNGB subunits are very similar. Nevertheless, there is a smaller number of protein residues involved in the interactions with cGMP in the CNGB-type subunits of rod and cone, and the MM/GBSA energies are weaker than those in the CNGA-type subunit. This can be explained by the different conformations of C-helix and β7-αP loop in the CNBD of the CNGB compared to the CNGA subunit^6,31^ as described above. However, the same set of amino acid residues compared to CNGA-type subunits are involved in cGMP binding according to the MM/GBSA analysis, e.g., R1062 (αP-β7 loop), T1043 (αP-β7 loop), and S1032 (αP) in rod CNGB1a, and R604, T605, and S594 in cone CNGB3, respectively.

The total binding free energies of cGMP in the rod and cone CNGA subunit structures computed with MM/GBSA method^33,34^ are nearly identical (−78.1 kcal/mol in rod CNGA1, −78.7 kcal/mol in cone CNGA3). In CNGB subunits, these numbers are −69.3 and −40.8 kcal/mol in the rod and cone structures, respectively, which suggests weaker binding due to the inactivated conformation of the C-helix. In addition, the docking scores obtained with the RosettaLigand software^35,36^ strongly suggest that cGMP has the same binding affinity to rod and cone CNGA subunits, which yielded docking scores of −13.3 and −13.9 Rosetta energy units (REU), respectively. Docking of cGMP in CNGB subunits yielded slightly weaker scores of −12.8 and −11.5 REU in rods and cones, respectively, which reflect a less efficient binding to CNGB-type subunits compared to that of CNGA-type subunits. Overall, docking results are also in good agreement with the experimental data, yielding RMSD values of the top scored pose of cGMP after docking in relation to the cryo-EM pose of cGMP from 0.45 to 0.60 Å (CNGA1: 0.49 Å, CNGB1a: 0.45 Å, CNGA3: 0.60 Å, CNGB3: 0.45 Å).

### Characterization of cGMP-Induced Gating in Rod and Cone CNG Channels

**Figure 2:**
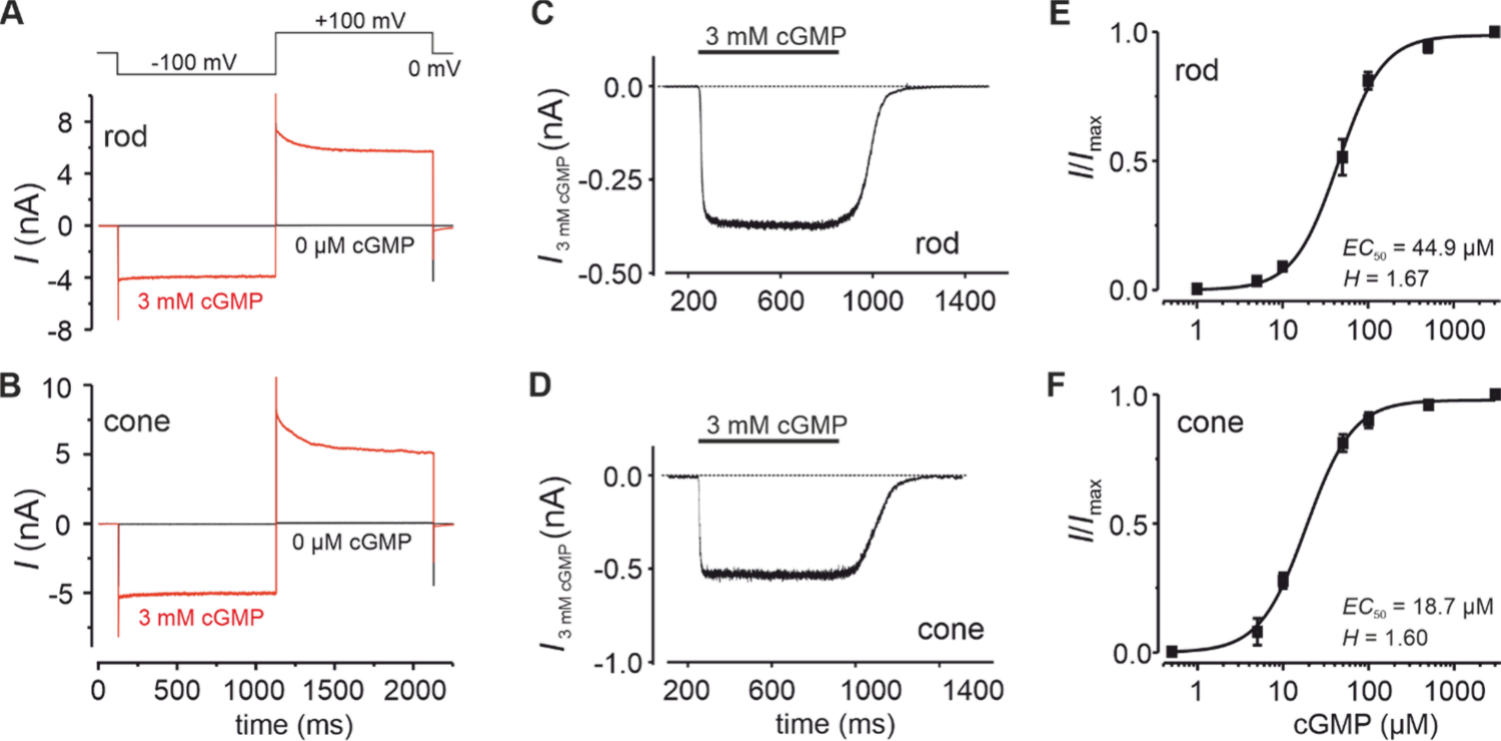
(A, B) Representative rod and cone CNG-channel currents in the presence (red) and absence (black) of cGMP. The voltage protocol is depicted on top of the diagrams. For this analysis, the current amplitude at the end of the +100 mV pulse was used. (C, D) Mean activation and deactivation time courses for rod and cone CNG channels measured by applying fast concentration jumps from 0 to 3 mM cGMP and back to 0 μM cGMP (*n* = 5 and 6 for rod and cone, respectively). The respective activation (τ_act_) and deactivation (τ_deact_) time constants were obtained by fitting the respective mean traces with monoexponential function eq 1: τ_act_ = 5.46 ms and τ_deact_ = 55.6 ms for cone CNG channels and τ_act_ = 8.96 ms and τ_deact_ = 47.52 ms for rod CNG channels. (E, F) Concentration–activation relationships for rod and cone CNG channels. The experimental data points, representing means of several experiments (*n* = 6–8 for rod and 5–9 for cone), were fitted with eq 2, yielding the *EC*_50_ and *H* values (see also Table S1). Error bars indicate ± SEM.

For the electrophysiological characterization, retinal CNG channels were heterologously expressed in *Xenopus laevis* oocytes, and their cGMP-induced activation was studied by means of patch-clamp recordings using the inside-out patch configuration. The presence of heterotetrameric CNG channels, composed of both CNGA- and CNGB-type subunits, in the oocyte plasma membrane was confirmed in parallel studies by means of several tests (e.g., cAMP-induced activation, l-*cis*-diltiazem-induced block and colocalization experiments between a plasma membrane fluorescent marker and TFP-labelled channels).^20,23^ The cAMP-induced current and the extent of l-*cis*-diltiazem-induced current block had similar characteristics as observed for native heterotetrameric CNG channels.^37−39^Figure 2 shows representative rod and cone CNG-channel currents in the presence of cGMP (Figure 2A,B). The gating kinetics, studied by applying fast cGMP concentration jumps, was similar for rod and cone CNG channels and was up to ∼9 and ∼60 ms for the activation and deactivation time courses, respectively (Figure 2C,D, see also Materials and Methods). We next determined the apparent affinity of rod and cone CNG channels measured in the presence of cGMP by approximating the Hill function (eq 2) to the relative current amplitudes plotted against different ligand concentrations. The apparent affinity of rod channels was approximately 2.4 times smaller than that of cone channels (44.9 vs 18.7 μM cGMP, Figure 2E,F and Table S1). The Hill coefficient (*H*), indicating the degree of cooperativity between subunits during channel gating, was similar for both CNG-channel isoforms (1.67 for rod vs 1.60 for cone CNG channel).

The outcomes of our computational data lead to the conclusion that no notable differences exist in the interaction pattern of cGMP with both CNGA-type and CNGB-type subunits of rod and cone CNG channels, respectively. This can be due to the high amino acid sequence identity of ∼79% of rod and cone CNGA-type subunits and ∼68% of rod and cone CNGB-type subunits. Within the CNBD itself, the sequence identity is even higher, with only 11 out of 121 amino acids differing between the CNBD of rod and cone CNGA-type subunits and 25 out of 117 amino acids differing within the CNBD of rod versus cone CNGB-type subunits (Figure 1D). The mismatched positions are not in close vicinity to the cGMP binding pocket and therefore fail to introduce selective interactions with cGMP. However, the experimental patch-clamp data reveal a clear difference in the apparent affinity of rod and cone CNG channels. This suggests that although the ligand-binding modes are similar (Figure 1E,F), differences in the binding and gating following the first binding event might contribute to the observed rod- and cone-specific apparent affinities.

### None of the Tested cGMP Modifications Selectively Inhibited Rod CNG Channels

**Figure 3:**
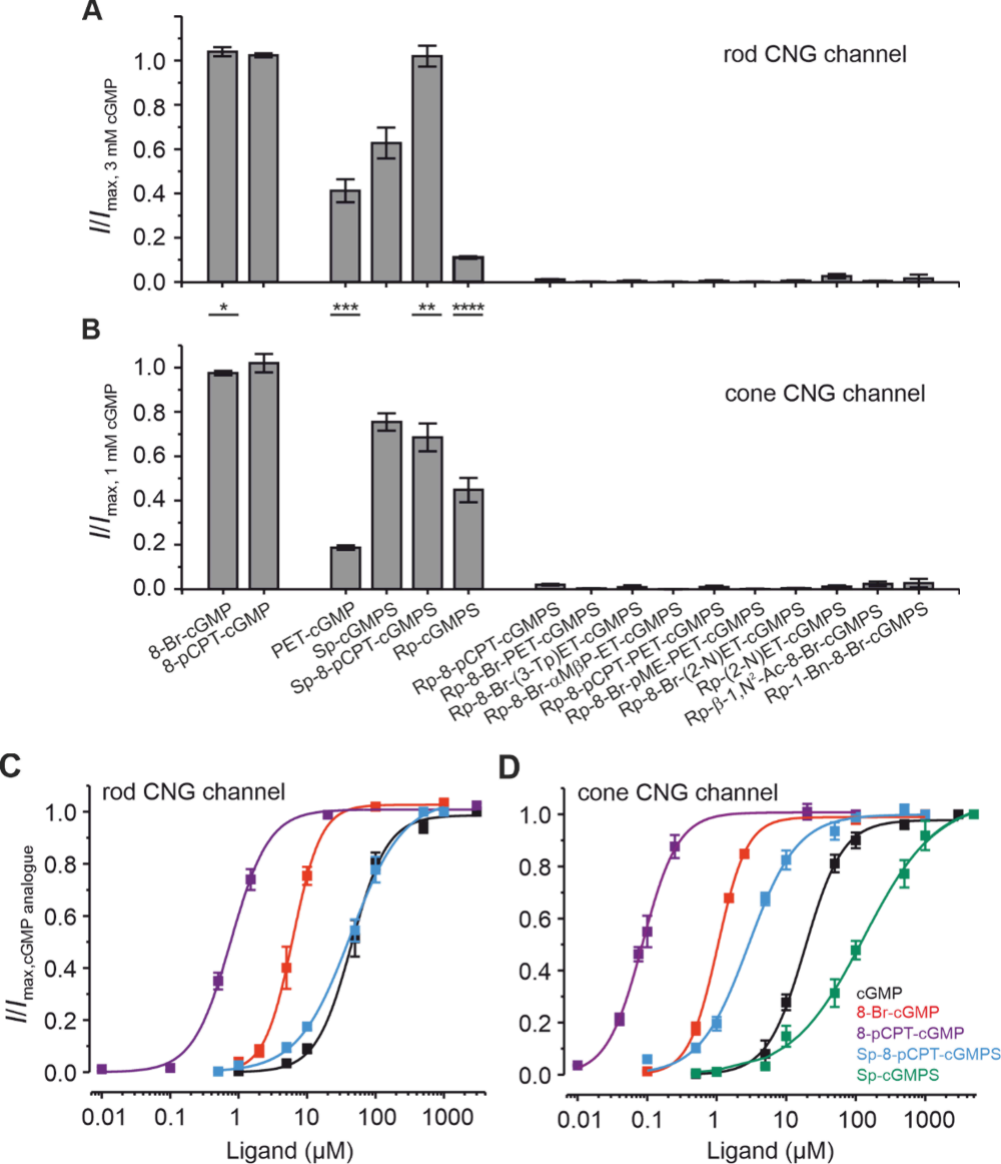
Efficacy and potency of cGMP analogues to activate rod and cone CNG channels. (A, B) Efficacy of tested cGMP analogues to activate rod (A) and cone (B) CNG channels. Maximal channel activation induced by the respective cGMP analogues (for GMP-analogues concentrations see Table S2) was related to current amplitude in the presence of saturating cGMP (3 and 1 mM for rod and cone CNG channels, respectively; *n* = 4–14 for rod and 5–18 for cone). Error bars indicate ± SEM. The statistical analysis is shown in Table S2. (C, D) Concentration–activation relationships for rod and cone CNG channels in the presence of cGMP (black line and symbols), 8-Br-cGMP (red line and symbols), 8-pCPT-cGMP (violet line and symbols), Sp-8-pCPT-cGMPS (blue line and symbols), and Sp-cGMPS (green line and symbols; for cone only). Data points representing means of several experiments were fitted with eq 2. The obtained *EC*_50_ and Hill coefficients (*H*'s) are included in Table S1. For Sp-cGMPS with rod CNG channels, measurements for the concentration–response relationship could not be concluded because of the lack of reaching saturating channel activity even in the presence of 10 mM.

In our quest to identify selective modulators for either rod or cone CNG channels, we turned our attention to cGMP analogues. To correlate structural features of the cGMP analogues with their binding mode and their induced effect on retinal CNG channels, we included in our analysis cGMP analogues that lead to a similar activation of the channels as cGMP, compounds that behaved as partial agonists, and compounds that triggered no channel activation at all. Earlier reports on the effects of some of these analogues on CNG channels showed no rod vs cone selectivity, or no direct comparison between different CNG-channel isoforms was performed.^19,40−42^ We tested 16 cGMP analogues with modifications to the C8 position of the guanine ring, the cyclophosphate, or the N1 and N^2^ positions of the guanine ring (Table 1). We measured first the maximal current through the CNG channels in the presence of saturating concentrations of the respective cGMP analogues. In a second step, the cGMP analogue-induced current was related, within the same patch, to the cGMP-induced current (Figure 3). Based on their effect on CNG channels, these analogues were classified for analysis purposes as “effective”, “partially effective”, and “ineffective”.

Only 8-Br-cGMP and 8-pCPT-cGMP, both with substitutions at position C8 of the guanine ring, were very effective in activating the retinal CNG channels. Among these, 8-Br-cGMP exhibited slightly higher efficacy in activating rods compared to that of cones. The next category of compounds, which includes PET-cGMP, Rp-cGMPS, Sp-cGMPS, and Sp-8-pCPT-cGMPS, was only partially effective when activating cone CNG channels (Figure 3A,B). PET-cGMP presents substitutions at positions N1 and N^2^ of the guanine structure, whereas the other compounds of this group are phosphorothioate derivatives of cGMP.^43^ With the exception of Sp-8-pCPT-cGMPS, which was very effective when activating rod CNG channels, all compounds of this group (up to 5 mM, see also Materials and Methods) lead only to a partial activation of these channels (∼41.2, ∼11, and ∼62.8% for PET-cGMP, Rp-cGMPS, and Sp-cGMPS, respectively). Significant differences between the activation levels of rod vs cone CNG-channels were observed with all compounds of this group, apart from Sp-cGMPS (Figure 3; for statistics, see Table S2). Among these, the highest selectivity for cone over rod was registered for Rp-cGMPS (∼43.6 and 11% cone vs rod CNG-channel activation). Based on these results and on those of Vighi et al., who reported an efficient delay of retinal degeneration in the presence of Rp-8-Br-PET-cGMPS,^19^ we selected for our study 10 further Rp-modified cGMP analogues (Table 1). The phosphorothioate modification of the cyclic phosphate was combined with different chemical substitutions in an attempt to increase the chances of achieving the CNG-channel isoform selectivity. The patch-clamp experiments indicate that none of the tested Rp-modified cGMP analogs exhibit selectivity in favor of rod over cone CNG channels. (Figure 3A,B). Moreover, neither of them could trigger a significant activation of both CNG-channel isoforms and were therefore labeled as “ineffective”.

For the effective and partially effective cGMP analogues, we also determined their potency when activating the retinal channels. For this, full concentration–activation relationships were determined (Figure 3C,D), which were fitted by eq 2, yielding the concentration of half-maximum activation (*EC*_50_) and the Hill coefficient (*H*) (see also Table S1). In agreement with previous results,^20^ compared to cGMP, the potency in activating the channels was increased by ∼7- and ∼18-fold with 8-Br-cGMP and by ∼58- and 234-fold with 8-pCPT-cGMP for rod and cone CNG channels, respectively (see also Table S1). The potency of Sp-cGMPS was comparable to that of cGMP when activating rod channels but ∼6-fold lower when activating the cone channels. Sp-8-pCPT-cGMPS was less potent than cGMP when activating both CNG channel isoforms. For the other two compounds of this group, PET-cGMP and Rp-cGMPS, the lack of significant channel activation for either cone or rod CNG channel, respectively, did not allow a correct determination of the *EC*_50_ values.

Based on the electrophysiological data, we can conclude that analogues with substitutions at C8 of the guanine moiety (R1 group in Table 1) mainly have a similar efficacy but much better potency as cGMP when activating retinal CNG channels. Analogues with Rp- and Sp-modifications at the cyclic phosphate moiety of cGMP (R2 group), as well as analogues with substitution at N1 and N^2^ (R3/R4 group), have weaker efficacy than cGMP for both channel isoforms. Combination of R1 + R2, as well as combination of R2 + R3, R1 + R3/R4, or R1 + R2 + R3/R4, results also in analogues with weaker efficacy than cGMP. Notably, only R1 + R2_(Sp-)_ had a similar efficacy as cGMP for rod channels but a weaker one for cone channels. This may be due to the fact that the R2_(Sp-)_-containing analogue showed a potency lower than that of cGMP when activating cone CNG channels. The decrease in potency could be rescued when combining R2_(Sp-)_ with the R1 group so that analogues with R1 + R2_(Sp-)_ substitutions (e.g., Sp-8-pCPT-cGMPS) present a higher potency than cGMP when activating cone CNG channels.

### None of the Tested cGMP Analogues Showed Selective Binding Scores

For a better understanding of the observed differences in channel activation, we analyzed next the structural and energetic basis of binding of different cGMP analogues to rod and cone CNG channels using ligand docking, MD simulations, and MM/GBSA analysis, as described for cGMP. We compared the binding poses of cGMP analogues with each other and with that of cGMP and calculated the root-mean-square deviation (RMSD) of the ligand atom positions. Overall, we found that the binding modes of all cGMP analogues to rod vs cone CNG channels are very similar. Compared to cGMP, RMSD values were in the range of 0.3 to 0.8 Å for the rod CNGA1 subunit, with higher RMSD values observed for analogues with bulkier substituents, such as thiophene, phenyl, and naphthalene groups at positions N1, N^2^ in analogues (e.g., Rp-8-Br-(3-Tp)ET-cGMPS, Rp-8-Br-PET-cGMPS, and Rp-8-Br-(2-N)ET-cGMPS, respectively). The RMSD values found in case of the cone CNGA3 subunit, as well as in case of the CNGB subunit structures of rod and cone CNG channels, were also in the range of 0.3–0.8 Å, increasing in the direction of cGMP analogues with bulkier substituents.

**Figure 4:**
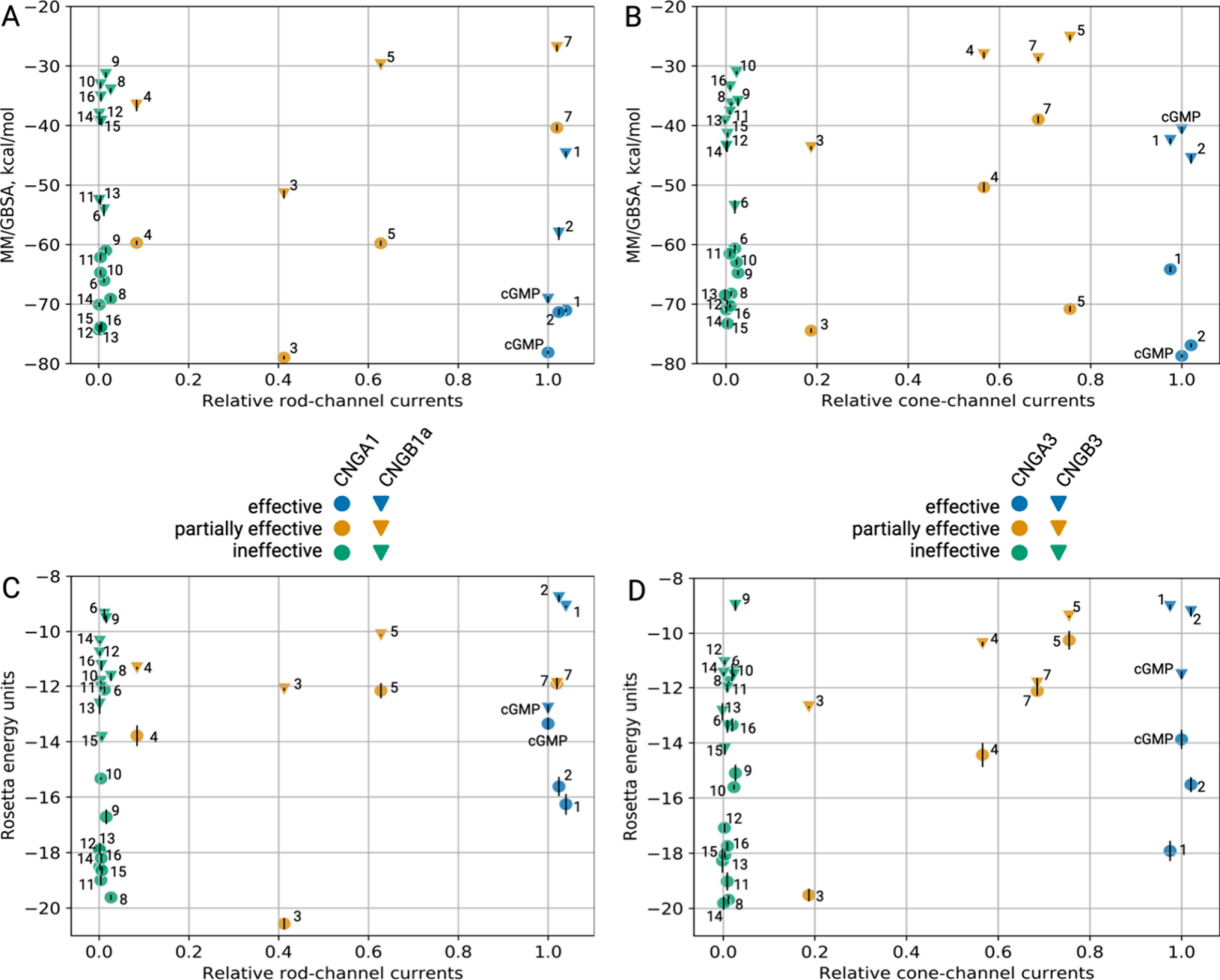
Relation of MM/GBSA energies or docking scores to relative CNG-channel currents induced by cGMP and different cGMP analogues. (A, C) CNGA1/CNGB1a subunits. (B, D) CNGA3/CNGB3 subunits. **1:** 8-Br-cGMP, **2:** 8-pCPT-cGMP, **3:** PET-cGMP, **4:** Rp-cGMPS, **5:** Sp-cGMPS, **6:** Rp-8-pCPT-cGMPS, **7:** Sp-8-pCPT-cGMPS, **8:** Rp-(2-N)ET-cGMPS, **9:** Rp-1-Bn-8-Br-cGMPS, **10:** Rp-β-1-N^2^–Ac-8-Br-cGMPS, **11:** Rp-8-Br-(3-Tp)ET-cGMPS, **12:** Rp-8-Br-PET-cGMPS, **13:** Rp-8-Br-αMβP-ET-cGMPS, **14:** Rp-8-Br-pMe-PET-cGMPS, **15**: Rp-8-Br-(2-N)ET-cGMPS, and **16**: Rp-8-pCPT-PET-cGMPS.

The computational docking scores and MM/GBSA energies of the cGMP analogues were compared to the level of activation that these compounds triggered with rod or cone CNG channels. Figure 4 illustrates the relation between MM/GBSA binding energies and the compounds’ activity value, represented as relative current induced by high concentrations of the respective cGMP analogues compared to the current elicited by saturating cGMP (*I*/*I*_max_). Each group of compounds (effective analogues in blue, partially effective analogues in yellow, and ineffective analogues in green) spans a rather broad range of MM/GBSA energies and docking scores (Figure 4). Furthermore, MM/GBSA and Rosetta energy values of CNGB1a and CNGB3 are overall weaker than that of CNGA-type subunits in both rods and cones. This reflects the differences in the C-helix up and down conformations in CNGA and CNGB subunits, respectively. The MM/GBSA energies and docking scores are estimates of the ligands’ binding strength, with more negative values suggesting stronger binding. Both metrics are highly correlated with each other for cGMP and cGMP analogues acting on CNG channels (Figure S4) (Pearson correlation coefficients of 0.79 and 0.75 for rod CNGA1 and cone CNGA3 structures, respectively), corroborating the *in silico* predictions. A higher ligand binding affinity could lead to a higher residence time in the binding pocket and stronger activation of CNG channels. However, we could not find any correlation between the MM/GBSA energies or docking scores with the ligand-induced channel activity (i.e., relative current values) (Figure 4). The lack of a distinct correlation between the *in silico* predicted binding energies of cGMP and its analogues and their biological activity data might arise from additional factors influencing the open probability and current amplitude through CNG channels beyond ligand binding affinity. In this regard, experimental determination of the binding affinity of the cGMP analogues on CNG channels would be needed, which could be helpful to further differentiate ligands by their physicochemical properties. However, measuring ligand binding, for example, by means of confocal patch-clamp fluorometry, as we previously did for the olfactory CNG channel and the HCN channel,^44,45^ is technically very challenging because fluorescently labeled cGMP analogues must be used. This involves adding a fluorescent probe to the already bulky cGMP-analogue molecule. Furthermore, for each cGMP analogue, it must be ensured that the fluorescent label does not have any side effects, such as influencing the binding affinity or channel gating by itself.

### cGMP Analogues with Substitutions at Position C8 of Guanine, Although Slightly Decreasing the Binding Energy Magnitude, Behave as Effective Ligands of CNG Channels

**Figure 5:**
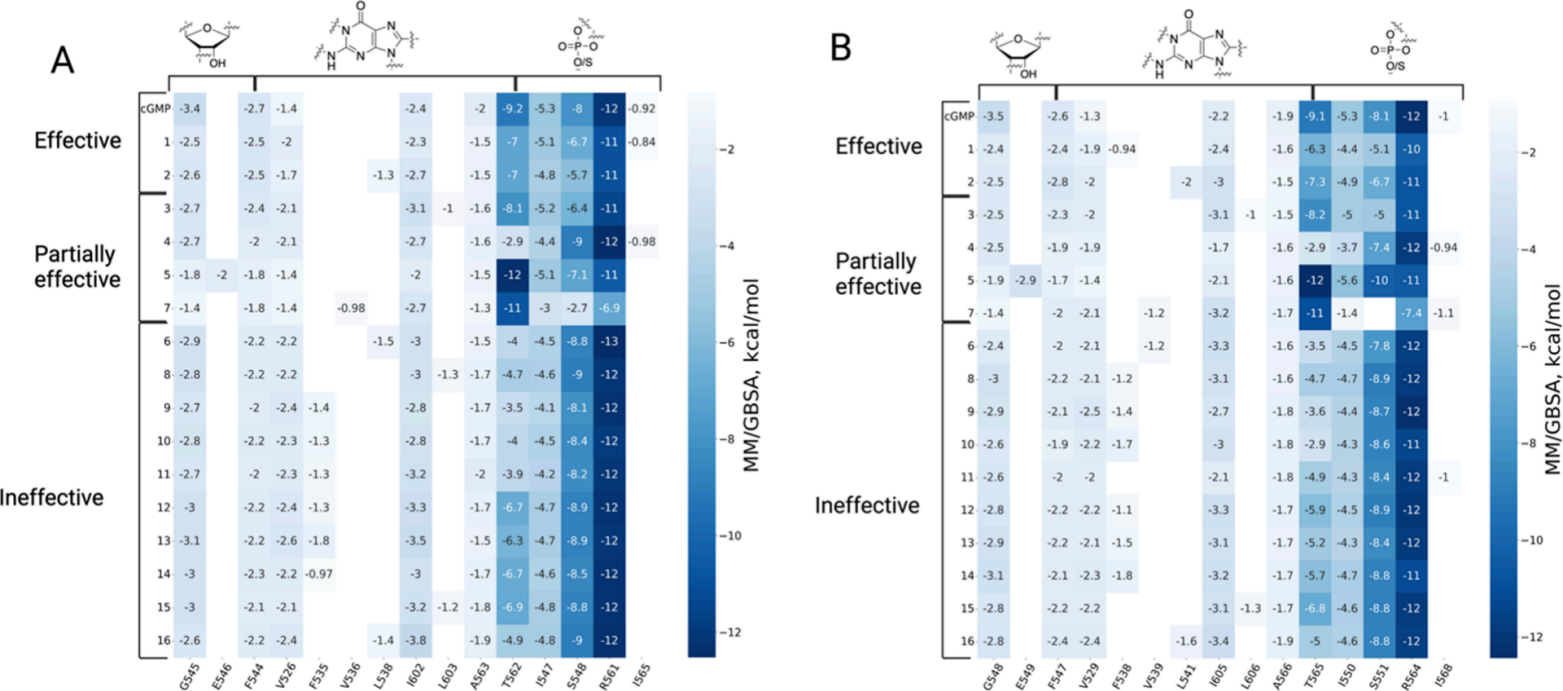
Heatmaps of per-residue contributions to the MM/GBSA binding energy of cGMP and cGMP analogues on CNG channels. (A) Per-residue energy breakdown results for the rod CNGA1 subunit. (B) Per-residue energy breakdown results for the cone CNGA3 subunit. The rows and columns of the heatmap contain the energy values of different ligands and protein residues, respectively. On top of the heatmap, the ligand moieties that interact with the protein residues in the heatmap are shown.

As observed from the electrophysiological experiments, the cGMP analogues with substitutions at position C8 of the guanine nucleobase behave as ligands with full efficacy (i.e., 8-Br-cGMP and 8-pCPT-cGMP) for both rod and cone CNG channels (Figure 3). The key interactions and the residues that contribute most to ligand binding based on the MM/GBSA energy and contact number analyses are highlighted in Figure 5 and Figures S1–S3.

**Figure 6:**
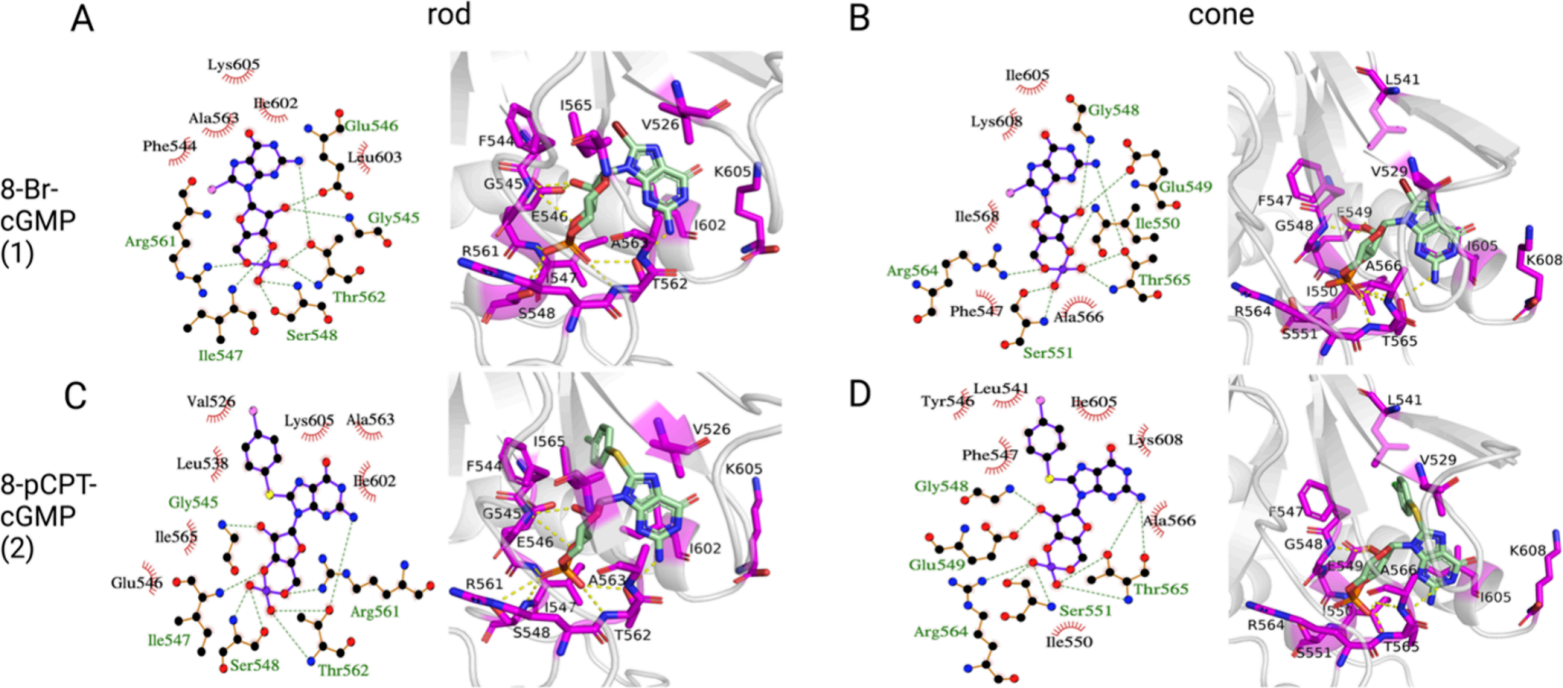
Binding modes of cGMP analogues with guanine ring C8 substitutions in CNGA-type subunit’s CNBD. For each ligand (A, B: 8-Br-cGMP and C, D: 8-pCPT-cGMP), 2D and 3D diagrams of their binding modes in the CNBD of rod (left) and cone (right) CNGA-type subunit are shown. Hydrophobic contacts are represented as red arcs in the 2D diagrams; hydrogen bonds are depicted as green dashed lines between amino acid residues and the ligand. Residues, important for binding, as shown by their MM/GBSA energy, are colored magenta in the 3D diagrams.

Figure 6 illustrates the binding poses of these cGMP analogues to the CNBD of the CNGA-type subunits. The respective binding poses to the CNGB-type subunits are provided in the Supporting Information (Figure S5).

8-Br-cGMP and 8-pCPT-cGMP have very similar binding modes (Figure 6). The phosphate or phosphorothioate group of the molecules makes hydrogen bonds to S548 (αP), R561 (αP-β7 loop), and T562 (αP-β7 loop) in rod CNGA1 and S551, R564, and T565 in cone CNGA3. These three residues make the largest contribution to the binding energy (Figure 5). T562 in rod CNGA1 and T565 in cone CNGA3 make additional hydrogen bonds with the amine group at the N^2^ position of the guanine ring. I547 (αP) is also involved in hydrogen bonding and steric interactions with the phosphate/phosphorothioate in CNGA1, whereas in CNGA3, this residue is I550 (Figure 6 B,D). The 2′-hydroxyl group of the ribose ring is hydrogen bonded to G545 (αP) and E546 (αP) in CNGA1 or to G548 and E549 in CNGA3. The bulky 8-*para*-chlorophenylthio substituent in 8-pCPT-cGMP leads to additional hydrophobic contacts with, e.g., L538 (β5) in CNGA1 and L541 in CNGA3 (Figure 6C,D). However, these interactions have only a minor contribution to the total binding energy (Figure 5).

The total free binding energy values calculated with the MM/GBSA method in the rod CNGA1 structure were −71.1 and −71.4 kcal/mol for 8-Br-cGMP and 8-pCPT-cGMP, respectively (Figure 4A, ligands 1 and 2). For the cone CNGA3 structure, a similar ranking of energy values was obtained: −64.2 and −76.9 kcal/mol for 8-Br-cGMP and 8-pCPT-cGMP, respectively (Figure 4B, ligands 1 and 2). Interestingly, none of these analogues is a better binder than the physiological ligand cGMP, which has the highest magnitude binding energy value among all ligands tested (−78.1 kcal/mol in CNGA1 and −78.7 kcal/mol in CNGA3).

Because of the inactivated conformation of the C-helix, the MM/GBSA energy values in CNGB-type subunits for effective analogues were worse, having −44.8 and −42.6 kcal/mol for 8-Br-cGMP in CNGB1a/CNGB3 subunits (Figure 4A,B, ligand 1) and −58.2 and −45.6 kcal/mol for 8-pCPT-cGMP in CNGB1a/CNGB3 subunits (Figure 4A,B, ligand 2), which were also the case for cGMP itself (−69.3 kcal/mol for CNGB1a and −40.8 kcal/mol for CNGB3). There is no clear preference for the rod over the cone CNG channel isoform among the cGMP analogues tested. This aligns with the electrophysiological assessments, where all the aforementioned compounds demonstrated comparable efficacy on both rod and cone CNG channels.

### cGMP Derivatives with Substitution at N1, N^2^ or Sp/Rp-Modifications and/or Substitution at C8 of the Guanine Group Behave as Partially Effective Analogues While Sharing a Similar Binding Mode with Effective Analogues

**Figure 7:**
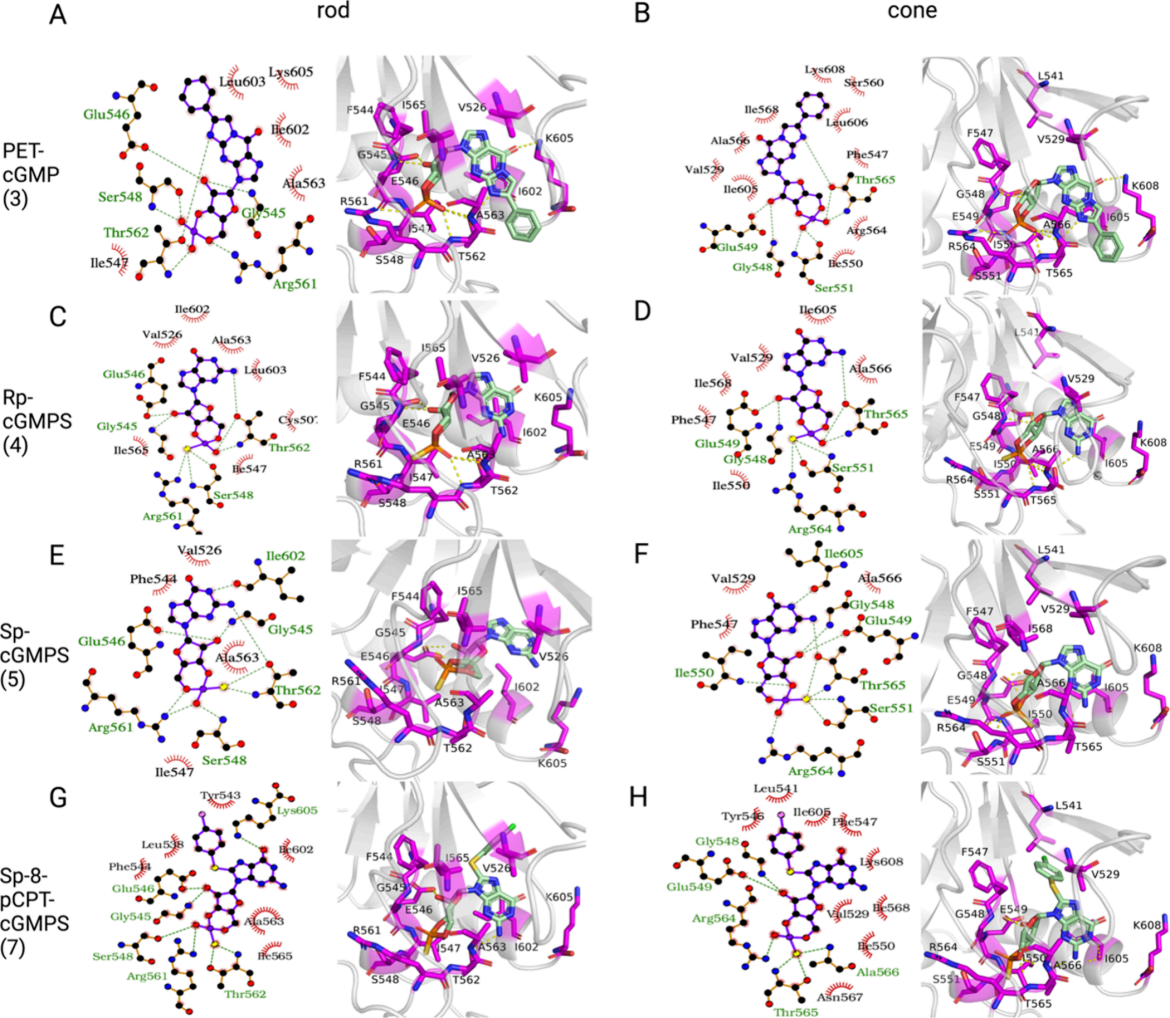
Binding modes of cGMP analogues with Rp-modification or substitutions at positions 1 and 2 of the nucleobase in the CNGA-type subunit. For each ligand (A, B: PET-cGMP; C, D: Rp-cGMPS; E, F: Sp-cGMPS, and G, H: Sp-8-pCPT-cGMPS), 2D and 3D diagrams of their binding modes in rod (left) and cone (right) CNG channels are shown. Hydrophobic contacts are represented as red arcs in the 2D diagrams; hydrogen bonds are depicted as green dashed lines between amino acid residues and the ligand. Residues, important for binding, are colored magenta in the 3D diagrams.

Figure 7 displays the binding modes and interactions of PET-cGMP, Rp-cGMPS, Sp-cGMPS, and Sp-8-pCPT-cGMPS in the rod and cone CNGA structures. The interaction modes in the CNGB-type subunit are shown in the Supporting Information (Figure S6). According to electrophysiological evaluations, PET-cGMP, Rp-cGMPS, and Sp-cGMPS are considered partially effective ligands for rod and cone CNG channels (see Figure 3). One exception is Sp-8-pCPT-cGMPS, which is an effective agonist of the rod CNG channel but a partially effective agonist of the cone CNG channel. Based on the observed interaction modes of these cGMP analogues (Figure 7), which are very similar to the ones of the full agonists displayed in Figure 6, the molecular reason for their lower activity on CNG channels could not be concluded. We confirmed MM/GBSA results with the more accurate but computationally more extensive thermodynamic integration (TI) method.^46^ We conducted TI energy calculations on 8-pCPT-cGMP, Sp-8-pCPT-cGMPS, and Rp-8-pCPT-cGMPS and observed that 8-pCPT-cGMP has a better binding energy than Rp-8-pCPT-cGMPS, which has a better binding energy than Sp-8-pCPT-cGMPS. The TI experiments confirmed the MM/GBSA energies and indicated the same energy order for the mentioned cGMP analogues.

The guanine ring of cGMP is replaced with a larger 1,N^2^-ethenoguanine ring in PET-cGMP with an additional β-phenyl substituent. The bulkier ring system leads to more, mostly hydrophobic contacts with residues at the end of the C-helix of the CNGA-type subunit, such as K605, L603 and I602 (αC) in rod CNGA1 and K608, L606, and I605 in cone CNGA3. The energetically most significant interactions are still those involving the phosphate or phosphorothioate group, such as hydrogen bonds with S548 (αP), R561 (αP-β7 loop), and T562 (αP-β7 loop) in CNGA1 (S551, R564, and T565 in CNGA3), as described for cGMP (see Figure 5) and cGMP analogues that act as full agonists (see Figure 6). Additional hydrophobic interactions involve residues V526 (β4), I547 (αP), and A563 (αP-β7 loop) in the rod isoform and V529, I550, and A566 in the cone structure. The predicted total binding free energies of PET-cGMP, Rp-cGMPS, Sp-cGMPS, and Sp-8-pCPT-cGMPS are −78.9, −59.7, −57.6, and −40.4 kcal/mol, respectively, in CNGA1 (Figure 4A, ligands 3, 4, 5, and 7) and −74.5, −50.4, −59.2, and −39 kcal/mol, respectively, in CNGA3 (Figure 4B, ligands 3, 4, 5, and 7). Energy values for CNGB-type subunits can also be found in Figure 4A,B. Thus, the Sp- or Rp- with phosphorothioate modifications or C8-substitutions of Rp-cGMPS, Sp-cGMPS, or Sp-8-pCPT-cGMPS lead to a drop of the binding energy magnitude calculated by MM/GBSA, whereas the magnitude of the binding energy of PET-cGMP is comparable to that of cGMP. The high degree of similarity in the types of interactions and the comparable binding energy of cGMP analogues with partial agonist behavior explains the lack of selectivity of these compounds for rod versus cone CNG channels.

### cGMP Analogues with Rp-Modifications at the Cyclic Phosphate Moiety of cGMP and Substitutions at N1, N^2^, and C8 Were Ineffective on CNG Channels and Span a Wide Range of Binding Energies

**Figure 8:**
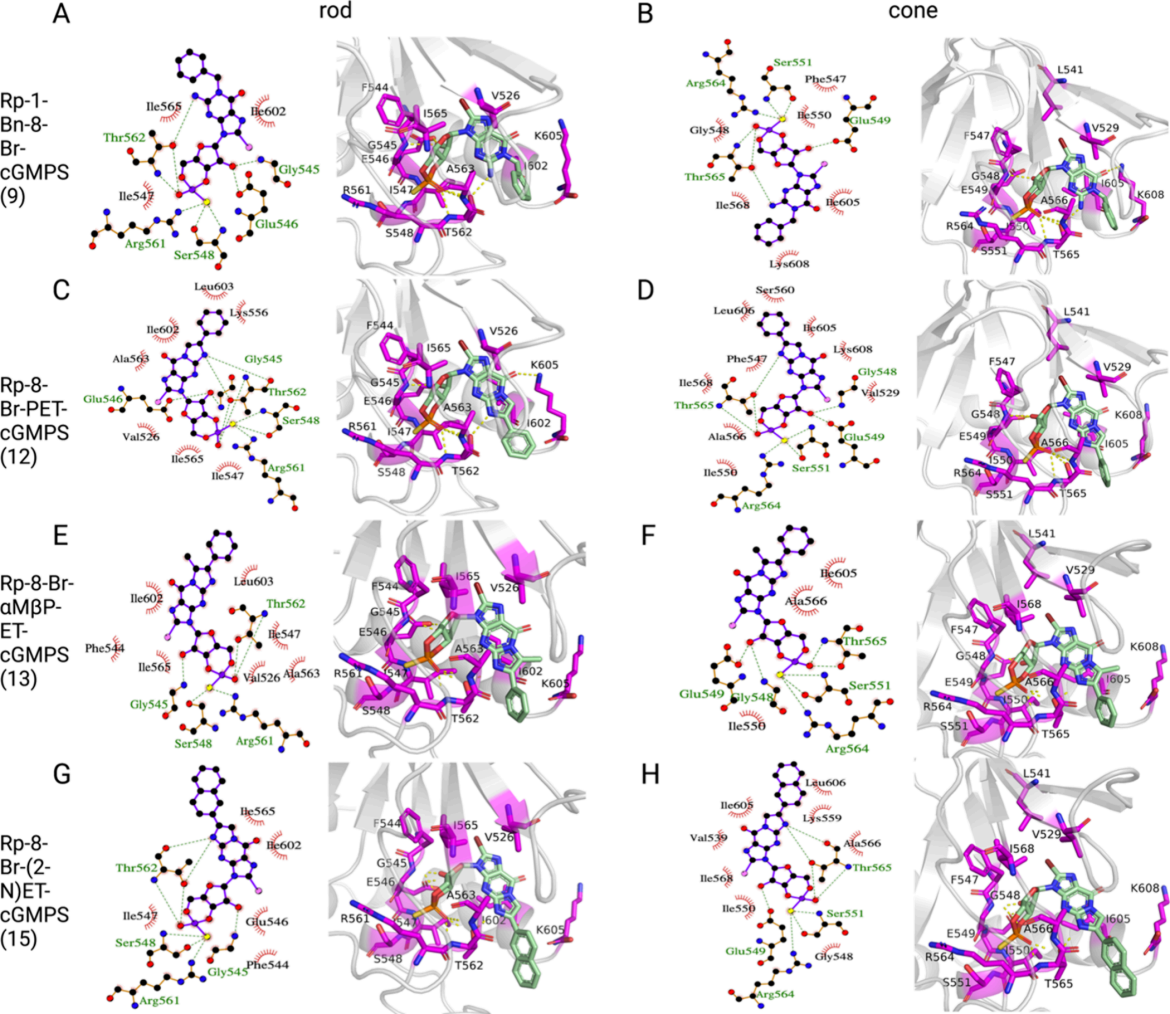
Binding modes of cGMP analogues with Rp-modifications at the cyclic phosphate moiety of cGMP and substitutions at N1, N^2^, and C8 in the CNGA-type subunit. For each ligand (A, B: Rp-1-Bn-8-Br-cGMPS; C, D: Rp-8-Br-PET-cGMPS; E, F: Rp-8-Br-αMβP-ET-cGMPS; G, H: Rp-8-Br-(2-N)ET-cGMPS), 2D and 3D diagrams of representative binding poses from the MD simulations in rod (left) and cone (right) CNG channels are shown. Hydrophobic contacts are represented as red arcs in the 2D diagrams; hydrogen bonds are depicted as green dashed lines between amino acid residues and the ligand. Residues, important for binding, are colored magenta in the 3D diagrams.

The remaining compounds from the set of tested cGMP analogs failed to elicit significant currents through CNG channels in the electrophysiological experiments and were considered ineffective analogues (see Figure 3). We analyzed the binding modes of these analogues and their interactions with the CNBD of CNGA- and CNGB-type subunits using docking and MD coupled to MM/GBSA calculations (Figure 8 and Figure S7). The molecular modeling results obtained on the CNGB-type subunits are provided in the Supporting Information (Figure S8). MM/GBSA binding free energies for this set of compounds vary from −75 to −55 kcal/mol in rod CNGA1 and −70 to −40 kcal/mol in cone CNGA3 (Figure 4A,B, ligands 6 and 8–16). Docking scores range from −21 to −12 Rosetta energy units (Figure 4C,D, ligands 6 and 8–16).

As for the cGMP analogues that behaved as partially effective and effective agonists (Figures 6 and 7), the interactions with the most significant energetic contributions are established by I547 (αP), S548 (αP), R561 (αP-β7 loop), and T562 (αP-β7 loop) in the CNGA1 structure and by I550, S551, R564, and T565 in the CNGA3 structure, involving hydrogen bonds with the phosphorothioate group. Additional hydrogen bonds are formed by the guanosine N^2^ atom with T562 (αP-β7 loop) in rod CNGA1 (T565 in CNGA3) and by the ribose 2′-hydroxyl group with G545 (αP) and E546 (αP) in CNGA1 (G548 and E549 in CNGA3).

A prominent feature of all analogues that were identified as ineffective ligands is larger substitutions of one to three rings that are joined to the guanine ring in positions N1 and N^2^. These larger substituents lead to interactions with residues in the C-helix of the CNBD, in addition to hydrophobic interactions with F544 (β6) and A563 (αP-β7 loop). For example, Rp-8-Br-PET-cGMPS can form hydrophobic interactions with I602 (αC) and L603 (αC) (I605 and L606 in cone CNGA3) (Figure 8C).

Whereas R561 (αP-β7 loop) in rod CNGA1 (R564 in cone CNGA3) has the largest energetic impact on binding for almost all cGMP analogues, T562 (T565) (αP-β7 loop) is the most important binding residue for two ligands: Sp-cGMPS and Sp-8-pCPT-cGMPS (compare with Figure 5). For S548 (S551) (αP) and R561 (R564), the trend is vice versa. These residues are less important for binding of Sp-cGMPS and Sp-8-pCPT-cGMPS according to the MM/GBSA analysis, which is opposite the rest of the ligands. Visual comparison of the binding complexes of Sp-cGMPS and Sp-8-pCPT-cGMPS with those of the other ligands suggests that the difference in the energetic contributions of R561 versus T562 is related to small changes in the distance between the phosphorothioate group and the side chains of R561 and T562 during the course of the MD simulation. Nevertheless, this atomistic structural change has no noticeable effect on the functional outcome because, as concluded from the patch-clamp experiments, both compounds are partially effective analogues. The same interaction pattern is observed in CNGB-type subunits, although with less strong binding energy values obtained with MM/GBSA and Rosetta docking calculations (Figure S2).

### Implications for a Better Understanding of Ligand Binding and Gating in Retinal CNG Channels

The MM/GBSA binding energies agreed well with Rosetta ligand docking scores but failed to correlate with CNG-channel activation for the series of cGMP analogues. This suggests that other factors, in addition to the ligand binding strength, influence the current flow through CNG channels. Our study sheds light on two critical processes defining how CNG channels function: (1) How can a similar ligand-binding mode result in different maximal effects and/or potencies? (2) How does gating influence ligand binding?

A straightforward approach would be to think in terms of different ligand efficacies, e.g., the ability of a ligand to induce conformational changes within the channel protein that result in channel activation. These gating changes are intricate, involving interactions specific to rod and cone subunits. In a recent study, even the lipid composition used in the experimental protocol had an impact on the conformational changes triggered by ligand binding.^47^ This suggests that the gating process is very dynamic, and therefore, subtle gating differences induced by changes in the energetic landscape due to the binding of different cGMP analogues cannot be excluded.

Another aspect that has to be considered is the dynamic nature of ligand-binding affinity during channel gating,^48^ whereas the binding affinities inferred from the docking experiments are static. The degree of activation induced by ligand binding depends not only on the affinity of the initial binding step to a closed channel but also on the affinity of the following binding steps mostly to an open channels. This complexity was highlighted by studies on homotetrameric CNGA2 and heterotetrameric CNGA2:A4:B1b concatenated constructs, which revealed subunit- and state-specific thermodynamics of ligand binding cooperativity.^29,49−51^ Experiments involving channel constructs containing subunits with disabled binding domains^32^ demonstrated that binding to the closed channel enhances affinity specifically for certain subunits, whereas channel opening enhances affinity for all subunits. Also for the structurally related HCN channels, Kusch et al. could describe an increase in binding affinity upon channel activation,^45^ thus proving the principle of reciprocity between ligand binding and gating in a receptor protein.^48^ Similarly, by means of binding measurements of fluorescence-labeled cAMP molecules to closed HCN2 channels and kinetic modeling, Kuschke and colleagues could show that the affinity of vacant cAMP binding sites escalates as the degree of occupancy rises.^52^ A similar analysis for the retinal channels, but also the availability of CNG-channel structures with different numbers of bound ligands, would definitely be of help in disentangling the intricate process of subunit cooperativity.

Notably, our study uncovered an interesting contrast to HCN channels, where N^6^-modified cAMP analogues, despite exhibiting different binding modes compared to the physiological ligand cAMP, activated the HCN2 channel with a similar efficacy as cAMP. This observation implies that the cumulative events following the initial binding reaction in HCN2 channels resemble those triggered by cAMP.^53^ This discrepancy between HCN and CNG channels could arise from their distinct primary activation mechanisms: HCN channels respond to voltage changes, whereas CNG channels rely on ligand binding. This might explain why CNG channels are more susceptible to even minor alterations in the ligand binding modes.

Different “resting states” of the channel protein can also influence ligand efficacy.^48^ The “resting state” refers to the channel’s spontaneous activity in the absence of ligand and depends on the different residual cGMP levels in rod and cone photoreceptors in the presence of light stimuli. Indeed, previous studies reported different levels of spontaneous activity for cone and olfactory CNGA channel isoforms.^54,55^

While this paper was in revision, Porro et al. presented a detailed mechanism by which an activated HCN channel can modulate its binding affinity.^56^ The authors showed that α-helices D and E, downstream of the CNBD, significantly increase ligand efficacy and affinity by interacting with the C-helix of the CNBD. Whether a mechanism similar to that of HCN channels, by which gating influences binding affinity, is also present in CNG channels remains to be determined.

Our results also highlight the importance of considering the physiological component when interpreting computational predictions in the context of ion channel biology. It is worth noting that the cryo-EM structures used in our simulations were acquired in the absence of CaM,^9^ whereas our electrophysiological studies were conducted on heterologously expressed CNG channels in *Xenopus* oocytes, likely in the presence of endogenous CaM.

## Conclusions

Beyond their contribution to the visual and olfactory sensory systems, CNG channels have been identified in many other tissues (e.g., brain, kidney, testis, liver, and adrenal gland).^1^ Unfortunately, their physiological roles within these organs are only poorly understood. Development of selective modulators will not only help in developing new therapies for existent channelopathies but also significantly accelerate the elucidation of the individual role of the CNG-channel isoforms within the different organs. Over the past decades, a great number of cGMP analogues have been synthesized. No systematic analysis of their effects on CNG-channel isoforms has existed so far. In this study, we examined by means of the patch-clamp technique the effect of 16 cGMP analogues on rod and cone CNG channels with the main goal of identifying specific inhibitors for rod CNG channels. We also investigated the molecular interactions between cGMP and cGMP analogues with the CNBD in rod and cone CNG channel structures by using molecular docking and molecular dynamics simulations coupled with MM/GBSA energy analysis.

**Figure 9:**
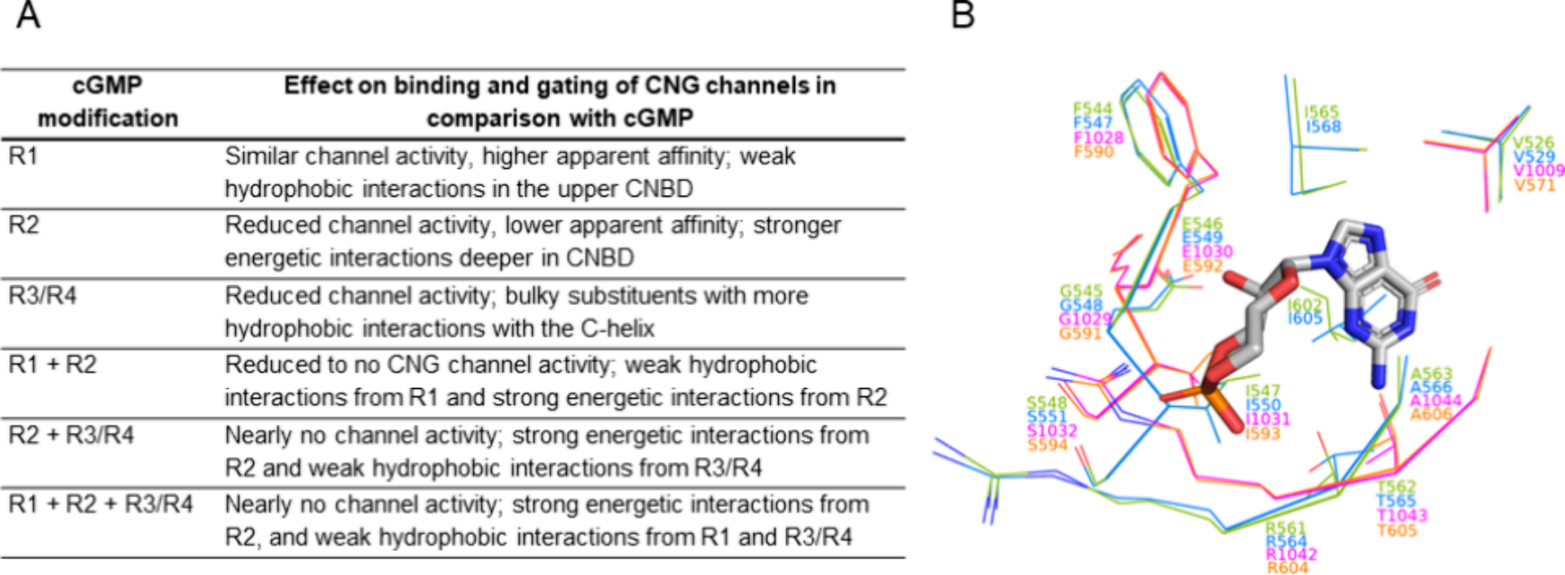
Binding modes of cGMP analogues in the CNBD of rod and cone CNG channels. (A) Structure–activity relationship of various cGMP modifications (for meaning of R1 to R4, see Table 1). (B) 3D alignment of cGMP and cGMP-coordinating residues (side chains) in CNBDs of CNGA1 (green), CNGA3 (blue), CNGB1a (magenta), and CNGB3 (orange) indicating identical ligand binding modes and interactions in all CNG-channel isoforms and subunits.

Unfortunately, our experimental results did not reveal a strong functional modulator for either the rod or cone CNG channel isoform. Nevertheless, some differences in rod vs cone channel activity were observed with the partially effective ligands. Whereas for PET-cGMP and Sp-8-pCPT-cGMPS the difference in the triggered activity with rod and cone CNG channels merely reached 2-fold (see Table 1), Rp-cGMPS showed in a previous study no influence on rod and cone CNG channels when coapplied with cGMP.^20^ One possible reason for the lack of selectivity is that the chemical substructures that are common to all ligands, i.e., the guanine ring, the ribose ring, and the phosphate or phosphorothioate group are oriented in the same manner in the respective CNBDs (Figure 9). Concordant binding modes are also observed when comparing the modeling results obtained for rod and cone CNG channel structures. F544 (β6 strand), G545, I547, S548 (all αP helix), R561, T562, A563 (all αP-β7 loop), and I602 (C-helix) are the most important interacting residues in the rod CNGA1 structure, having the largest contribution to the binding energy (see Figure 5). In the cone CNGA3 structure, the most important residues in terms of ligand binding are F547 (β6 strand), G548, I550, S551 (all αP helix), R564, T565, A566 (all αP-β7 loop), and I605 (C-helix). For cGMP analogues bearing bulky chemical modifications at the guanine and phosphate groups, additional interactions (e.g., with residues on the C-helix) become possible. This is reflected by a broad range of binding energies computed for cGMP derivatives, spanning approximately from −40 to −80 kcal/mol. But also these additional interactions did not increase the selectivity potential of any of the tested cGMP analogues for either rod or cone CNG channels.

Furthermore, when binding of cGMP and cGMP analogues to CNGA- versus to CNGB-type subunits was compared, no significant differences were observed. The most obvious differences were found in the conformation of the C-helix, which affects the ability of CNBD to make additional interactions with the cGMP ligands. Given that we employed the open I state structure of CNGB1a, characterized by the C-helix adopting a down-conformation, there are fewer interactions observed between the bound ligands and the C-helix. Nonetheless, we assume that utilizing the open II state structure would yield similar results. This assertion is based on our analysis, which suggests that the residues within the C-helix exert a minimal influence on the ligand binding energy. This conclusion finds support in our comparative per-residue decomposition analysis across both subunits. Although the CNBD amino acid sequence in CNGB-type subunit is only ∼35% identical to that of CNGA-type subunits, it is noteworthy that the residues involved in ligand binding (in β6 strand, αP helix, and αP -β7 loop) are conserved across all subunits in rod and cone isoforms (see Figure 9).

Our findings have important implications for understanding the roles of rod and cone cells in vision. The mechanisms that underlie the differences in the responses of these cells to light are not well understood, and our study sheds new light on this aspect. In particular, these results suggest that the differences in apparent affinity between rod and cone CNG channels are likely due to dissimilarities in their downstream gating mechanisms or influence of intracellular channel modulators, such as CaM for rods and CNG-modulin for cones, rather than differences in the ligand-binding domains and the respective ligand–CNBD interactions. Although additional research is necessary to fully elucidate the role of ligand binding in this context, it remains a promising option for therapeutic strategies for retinal degenerative diseases. Future studies may explore, for example, other ligand-binding pockets in CNG channels or pore blockers to achieve a selective modulation of CNG channel subtypes. Alternatively, an approach involving the differential regulation of downstream signaling pathways in either rod or cone photoreceptors presents another pathway worth exploring.

## Materials and Methods

### Animals

The procedures had approval from the authorized animal ethical committee of the Friedrich Schiller University Jena (UKJ 18-008 from 09.05.2018) and were carried out in accordance with §4 of the German animal protection law. Extreme efforts were made to reduce animal stress and to keep the number of frogs to a minimum.

### Heterologous Expression of Retinal CNG Channels

The subunits CNGA1 (NM_174278.2) and CNGB1a (NM_181019.2) from bovine rod photoreceptors and CNGA3 (NM_001298.2) and CNGB3 (NM_019098.4) from human cone photoreceptors were subcloned into the pGEMHE vector.^57^ The respective cRNAs were produced using the mMESSAGE mMACHINE T7 Kit (Ambion, Austin, TX, USA) after plasmid linearization with NotΙ.

*Xenopus laevis* oocytes were either harvested surgically under anesthesia (0.3% tricaine, MS-222, Pharmaq Ltd., Fordingbridge, UK) from female adults (Nasco, Fort Atkinson, USA) or purchased from Ecocyte (Castrop-Rauxel, Germany). These oocytes were first incubated for 90 min in Ca^2+^-free Barth’s medium containing collagenase A (3 mg/mL; Roche, Grenzach-Wyhlen, Germany) and (in mM) 82.5 NaCl, 2 KCl, 1 MgCl_2_, and 5 Hepes (pH 7.4). Afterward, they were injected with 50–130 ng cRNA encoding either for CNGA1/CNGB1a (1:4 ratio) or CNGA3/CNGB3 (1:2.5 ratio) channels. The injected oocytes were incubated at 18 °C for up to 6 days in Barth’s solution containing (in mM) 84 NaCl, 1 KCl, 2.4 NaHCO_3_, 0.82 MgSO_4_, 0.41 CaCl_2_, 0.33 Ca(NO_3_)_2_, 7.5 TRIS, cefuroxime (4.0 μg mL^–1^), and penicillin/streptomycin (100 μg mL^–1^) (pH 7.4). The vitelline membrane of the oocyte was manually removed before electrophysiological recordings.

### Electrophysiological Experiments

Rod and cone CNG-channel activity was recorded from inside-out patches of *Xenopus* oocytes by means of the patch-clamp technique. The patch pipettes (Hilgenberg GmbH, Malsfeld, Germany) were pulled from borosilicate glass tubing (outer diameter of 2.0 mm and inner diameter of 1.0 mm) or quartz tubing (outer diameter of 1.0 mm and inner diameter of 0.65 mm). The resistance of the solution-filled pipettes was 0.7–1.3 MΩ. The bath and pipette solution contained (in mM) 140 mM NaCl, 5 mM KCl, 1 mM EGTA, and 10 mM HEPES (pH 7.4). Recordings were performed by an Axopatch 200B amplifier (Axon Instruments, Foster City, CA, USA). Electrophysiological measurements were controlled by the Patchmaster software (HEKA Elektronik Dr. Schulze GmbH, Lambrecht, Germany). The sampling rate was 5 kHz, and the filter implemented in the amplifier was set to 2 kHz. For the concentration–activation relationships, the CNG-channel currents were elicited by voltage steps to −100 and to +100 mV. The holding potential was 0 mV. The gating kinetics of the CNG channels was recorded at −35 mV, the physiological voltage under dark conditions in photoreceptors. The test solutions were applied via a multibarrel device to the patches. Prior to the experiment, the concentrations of the respective dilutions were verified by UV-spectroscopy (Thermo Scientific NanoDrop 2000c Spectrophotometer, Waltham, USA). All experiments were carried out at room temperature.

### Quantifying Activation and Deactivation Time Constants

The kinetics of CNG-channel gating was studied by means of fast concentration jumps applied with a double-barreled θ-glass pipet mounted on a piezo-driven device, which was controlled by a software.^58^ The recording rate was 20 Hz. The effective solution switch time, which was previously determined with an open patch pipette and different solutions in the barrels, was negligible compared to the channel activation and deactivation time courses. The respective time constants were determined by fitting the respective current traces with single exponentials:
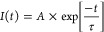
1where *A* is the amplitude, *t* is the time, and τ is the time constant for either activation or deactivation.

### Fitting Steady-State Concentration–Activation Relationships

Concentration–activation relationships were fitted with the Origin software using the Hill equation:
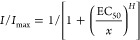
2where *I* is the actual current amplitude and *I*_max_ is the maximum current amplitude at saturating concentration of the respective cyclic-nucleotide analogue. *EC*_50_ is the concentration generating the half-maximum current, and *H* is the Hill coefficient. Errors are given as the mean ± SEM. “*n*” refers to the number of electrophysiological measurements. The individual measurements were performed on different oocytes, with a limit of two experiments per oocyte. Each type of experiment and every cGMP analogue tested involved a minimum of two batches of oocytes.

### cGMP Analogues

All cGMP analogues were diluted from stock solutions prepared shortly before the experiment. The dilutions were done according to the technical details provided by the manufacturers (Mireca Medicines GmbH, Tübingen, and Biolog GmbH & Co. KG, Bremen, Germany). For more information regarding the preparation of the cGMP analogues, see previously reported methods^19^ and US20190292214 (“New equatorially modified polymer linked multimers of guanosine-3′,5′-cyclic monophosphates”). All compounds are >98% pure by HPLC analysis.

### Structure Preparation

The structures of the human rod CNG channel in the closed, cGMP-free form (PDF: 7RH9)^6^ and in the open, cGMP-bound form (PDB: 7RHH)^6^ as well as of the human cone CNG channel in the closed, cGMP-free form (PDB: 7RHS)^27^ were downloaded from the Protein Databank (PDB). At the time the experiments were started, there was no available human cone CNG channel structure in the open form. Accordingly, a model of the human cone CNG channel in the open form (76% sequence identity to rod CNG channel) was created using the Swiss-Model homology modeling server.^59^ Only the structure of the cyclic nucleotide-binding domain (CNBD) of the CNGA-type and CNGB-type subunits of rod and cone CNG channels was used for the ligand docking and MD simulation experiments with the cGMP analogues. Additional control calculations using the full heterotetrameric rod or cone CNG channel structure were performed in the case of the cGMP ligand. Molecular structures of cGMP and cGMP analogues were built using the Marvin program (version 23.17.0; release year 2023, ChemAxon http://www.chemaxon.com) and geometry-optimized using the Open Babel program.^61^ Conformer libraries of cGMP and every cGMP analog used in ligand docking with Rosetta were generated using the BCL::ConformerGenerator method.^62^ Generation of Rosetta ligand params files was done as described previously.^63^ Assignment of Amber atom types of cGMP ligands and calculation of atomic charges with the AM1-BCC method were done using the Antechamber program.^64^

### Ligand Docking

Docking of cGMP and cGMP analogs was carried out with RosettaLigand^35,36,65^ through RosettaScripts.^66,67^ Rosetta ver. 3.12 was used for all calculations. Prior to docking, the ligand was superimposed with the coordinates of cGMP found in the open cGMP-bound rod (7RHH) structure. A scoring grid was created across the binding pocket, centered on the starting position of cGMP with a size of 15 × 15 × 15 Å. The maximum allowed translation of the ligand from its starting position was 7 Å. In the low-resolution stage, 500 Monte Carlo moves of the ligand with a maximum translation of 0.2 Å and a maximum rotation of 20° per step were performed. In the high-resolution stage, the *ligand.wts* scoring function was used, and six cycles of alternating protein side chain and ligand conformer packing followed by a final minimization of the protein–ligand interface were performed. A total of 500 docking models were generated for each CNG subunit structure and each cGMP or cGMP analog ligand. The 20 best-scoring docking models ordered by interface_delta_X score were analyzed for noncovalent protein–ligand interactions, and the average interface_delta_X of the 20 best models was compared to the percentage of current induced by cGMP analogues with respect to the current at 3 mM cGMP (%Δ*I*).

### Molecular Dynamics Simulation

MD simulations of the rod and cone CNBD structure bound to cGMP or 1 of 16 tested cGMP analogues were performed using Amber20. Additional MD simulations were performed for cGMP bound to the whole heterotetrameric structure of the rod or cone CNG channel. The ff19SB force field for proteins,^68^ the general Amber force field (GAFF)^69^ for ligand atoms, and the lidpi17 force field^70^ for lipid atoms were used.

The CNBD–ligand complex structure was surrounded by a cubic TIP3P water box with a thickness of at least 13 Å between any protein or ligand atom and the edge of the box. The charge of the system was neutralized by adding Na^+^ or Cl^–^ ions. The MD system containing the whole rod or cone CNG channel structure bound to cGMP and embedded in a membrane of ∼350 POPC molecules was built using the membrane builder tool of the CHARMM-GUI website.^71^ A TIP3P water layer containing 150 mM neutralizing KCl and extending 24 Å from the closest protein atom along the *Z* axis was added on either side of the membrane. In addition, two Ca^2+^ ions were placed in the channel selectivity filter at positions S1 and S2, inferred from the positions of Ca^2+^ in the human cGMP-bound open CNGA1 structure (PDF: 7LFX), whereas two water molecules were placed at positions S1 and S3. SHAKE^72^ bond length constraints were applied to all bonds involving hydrogen atoms. Nonbonded interactions were evaluated with a 10 Å cutoff, and electrostatic interactions were calculated by the particle-mesh Ewald method.^73^

The energy of each CNBD–ligand system was first minimized using a two-step minimization procedure: 20,000 steps minimization of water and ions and 20,000 steps minimization of the whole system. With protein and ligand atoms constrained to their minimized coordinates, the system was then heated from 0 to 298 K over 150 ps in the NVT ensemble with a step size of 2 fs. After changing to the NPT ensemble, the system was equilibrated at 298 K and a reference pressure of 1 bar for 1 ns with weak positional restraints (with a force constant of 1 kcal mol^–1^ Å^–2^) applied to protein backbone and ligand heteroatoms. Langevin dynamics with a collision frequency of 1 ps^–1^ and an integration time step size of 2 fs was used in these steps. Positional restraints on protein and ligand atoms were then removed, and the system was equilibrated for another 1 ns without Cartesian restraints. Production MD was conducted for 500 ns using constant pressure and periodic boundary conditions and Langevin dynamics. Three independent replicas were carried out for each cGMP analogue and CNBD structure (CNGA or CNGB subunit of rod or cone channel).

The MD system consisting of the whole CNG channel with cGMP was first minimized for 10,000 steps using steepest descent followed by 10,000 steps of conjugate gradient minimization. With protein, ligand, and lipid atoms restrained to their minimized coordinates, the system was heated to 298 K in the NVT ensemble over 150 ps. After changing to the NPT ensemble, restraints on lipids and protein side chain atoms were gradually removed over 1 ns, and the system was equilibrated for another 1 ns at 298 K with weak positional restraints (with a force constant of 1 kcal mol^–1^ Å^–2^) applied to protein Cα atoms and ligand heavy atoms. Production MD was conducted for 1 μs by using a step size of 2 fs, constant pressure periodic boundary conditions, anisotropic pressure scaling, and Langevin dynamics. Three independent replicas were carried out for each rod and cone CNG channel.

### MM/GBSA Energy Calculations

The binding free energy (Δ*G*_binding_) of cGMP and each cGMP analog bound in the CNBD and the per-residue contributions to Δ*G*_binding_ were computed in the MM/GBSA procedure^33^ using the *MMPBSA.py* program.^34^ Starting from the heated and equilibrated MD system, three 500 ns long simulations were conducted for each cGMP or cGMP analogue docking model using an integration time step of 2 fs, constant pressure periodic boundary conditions, and Langevin dynamics. Molecular conformations were sampled at 20 ps intervals from the first 25 ns of each MD simulation to compute the molecular mechanics energy and solvation free energies. The single trajectory mode was applied; i.e., snapshots of protein, ligand, and protein–ligand complex were taken from the same trajectory. The ionic strength of water was set to 150 mM. The entropic contribution to Δ*G*_binding_ was estimated by applying the quasi-harmonic approximation (QHA),^74^ and 10,000 conformations of the protein–ligand complex were used for this analysis.

## Supporting Information Available

Heatmaps of fraction of protein–ligand contacts and their per-residue contributions for cGMP and its analogues interacting with the CNGA- and CNGB-type subunits of retinal CNG channels; binding modes of different cGMP analogues in the CNBD of CNGA- and CNGB-type subunits; correlation plots of MM/GBSA energies versus Rosetta ligand docking scores of cGMP and its analogues; and tables summarizing the *EC*_50_ and *H* values for CNG channels in the presence of cGMP and its analogues, along with the statistical analyses elucidating the effects of various cGMP analogues on the activity of the respective channels (PDF)
