# Supplementary material for: Similar Binding Modes of cGMP Analogues Limit Selectivity in Modulating Retinal CNG Channels via the Cyclic Nucleotide-Binding Domain
Source: ACS Chem Neurosci. 2024 Apr 5;15(8):1652–68. doi: 10.1021/acschemneuro.3c00665 (PMC11027099; doi:10.1021/acschemneuro.3c00665)
Supplement: Supplementary file 1 — cn3c00665_si_001.pdf [file cn3c00665_si_001.pdf]

Similar binding modes of cGMP analogues limit selectivity in modulating retinal CNG channels via the cyclic nucleotide-binding domain

Authors: Palina Pliushcheuskaya[1*], Sandeep Kesh[2*], Emma Kaufmann[2], Sophie Wucherpfennig[2], Frank Schwede[3], Georg Künze[1,4,5#] and Vasilica Nache[2#]

Affiliations:

[1] Institute for Drug Discovery, Medical Faculty, University of Leipzig, 04103 Leipzig, Germany.

[2] Institute of Physiology II, University Hospital Jena, Friedrich Schiller University Jena, 07743 Jena, Germany.

[3] BIOLOG Life Science Institute GmbH & Co KG, 28199 Bremen, Germany.

[4] Interdisciplinary Center for Bioinformatics, University of Leipzig, 04107 Leipzig, Germany.

[5] Center for Scalable Data Analytics and Artificial Intelligence, University of Leipzig, 04105 Leipzig, Germany.

*These authors contributed equally to this work

[#]Corresponding authors:

Email: georg.kuenze@uni-leipzig.de and vasilica.nache@med.uni-jena.de

SUPPORTING INFORMATION

Supplementary Figures

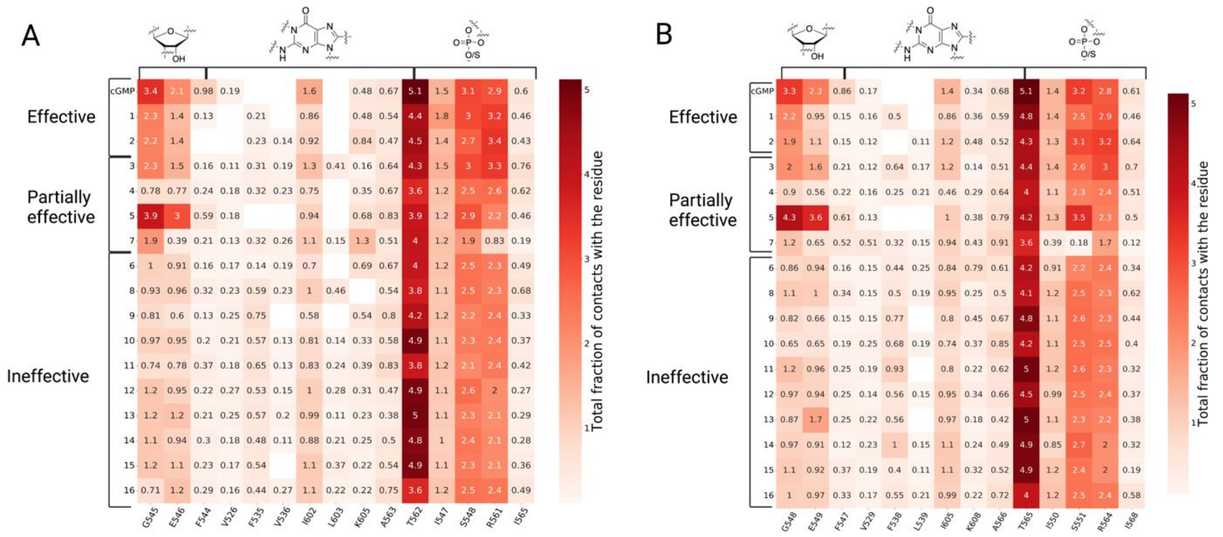

Figure S1. Heatmaps of fraction of protein-ligand contacts for cGMP and its analogues on CNG channels. A) Fraction of contacts for rod CNGA1 subunit. **B)** Fraction of contacts for cone CNGA3 subunit. The fraction of contacts corresponds to the number of heteroatom pair distances between the ligand and each protein residue below a cutoff distance of 3.5 Å normalized by the number of simulation frames.

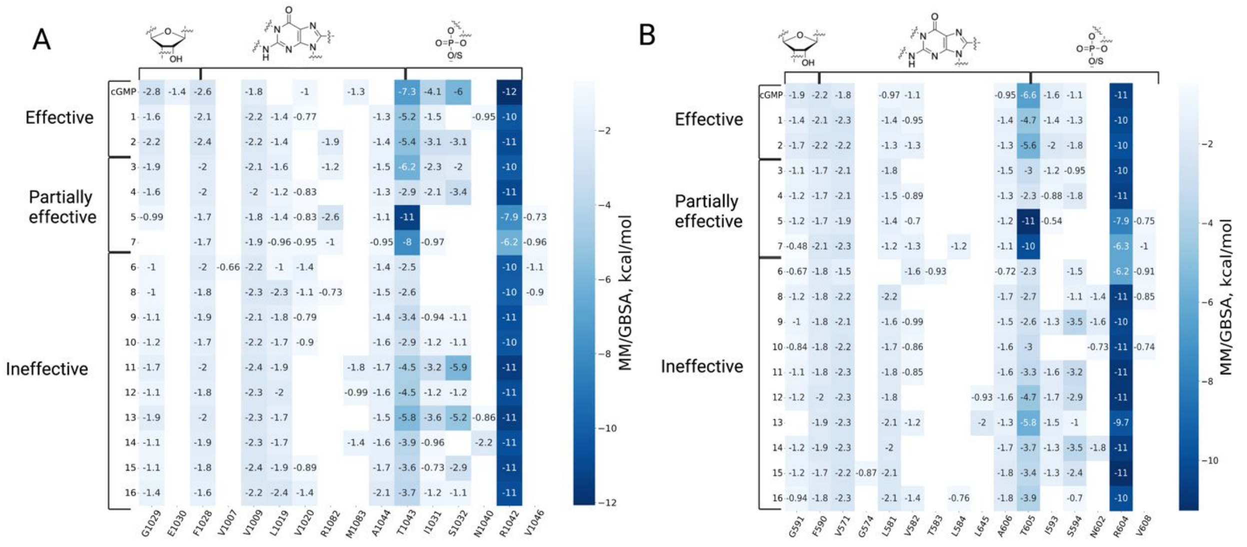

Figure S2. Heatmaps of per-residue contributions to the MM/GBSA binding energy of cGMP and its analogues interacting with the CNGB-type subunit of CNG channels. A) Per-residue

energy breakdown results for the rod CNGB1a subunit. **B)** Per-residue energy breakdown results for the cone CNGB3 subunit. The rows and columns of the heatmap contain the energy values of different ligands and protein residues, respectively. On top of the heatmap, the ligand moieties that interact with the protein residues in the heatmap are shown.

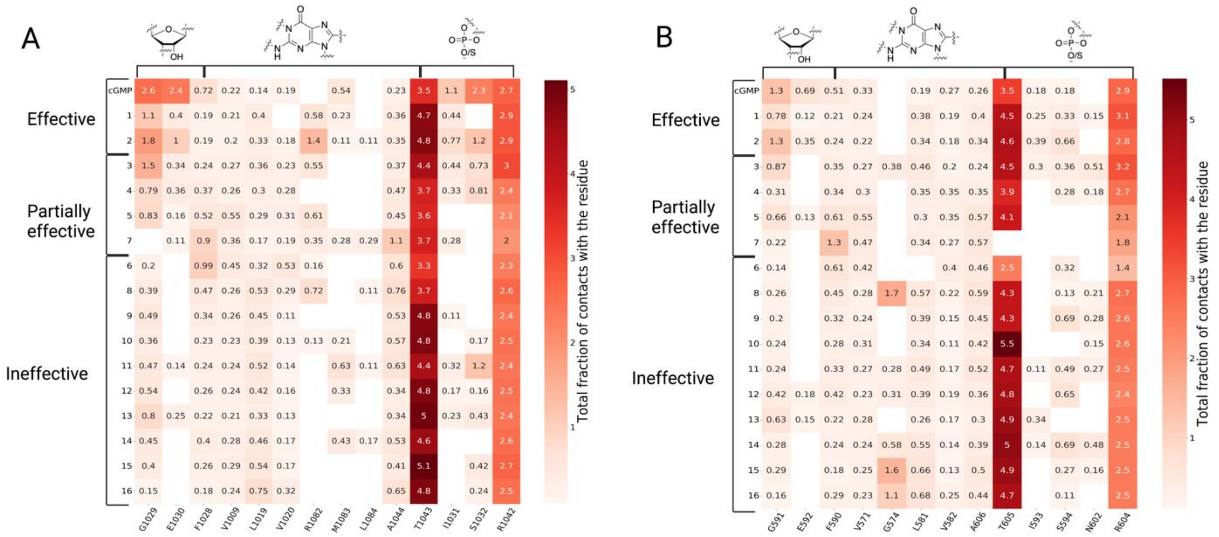

Figure S3. Heatmaps of fraction of protein-ligand contacts for cGMP and its analogues interacting with the CNGB-type subunit of retinal CNG channels. A) Fraction of contacts for rod CNGB1a subunit. **B)** Fraction of contacts for cone CNGB3 subunit. The fraction of contacts corresponds to the number of heteroatom pair distances between the ligand and each protein residue below a cutoff distance of 3.5 Å normalized by the number of simulation frames.

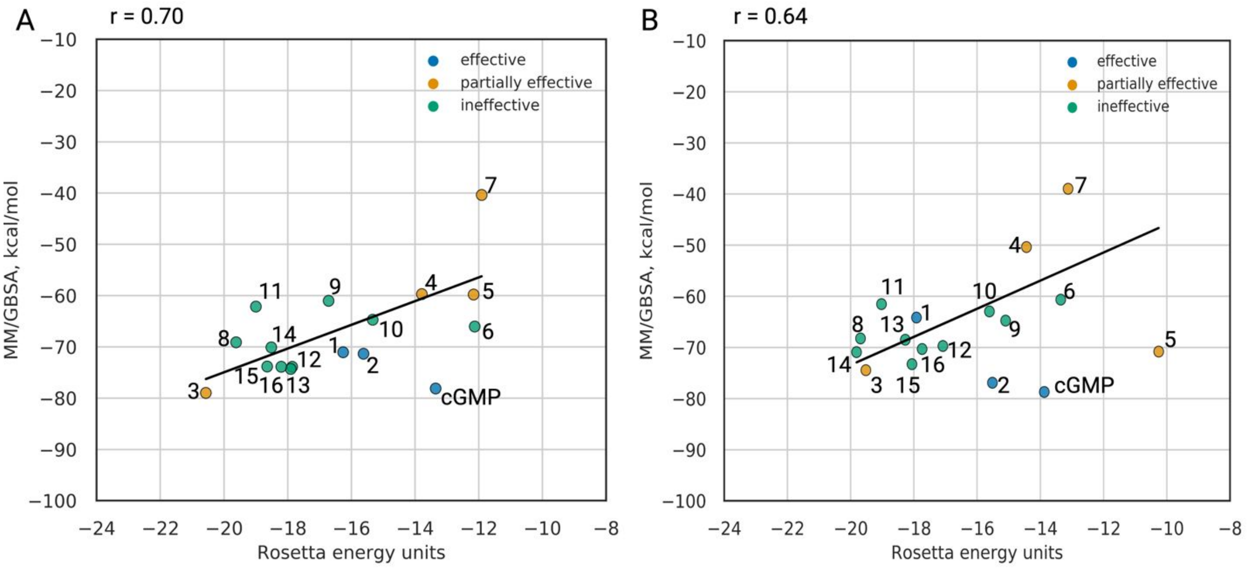

Figure S4. Correlation plots of MM/GBSA energies versus Rosetta ligand docking scores of cGMP and its analogues. A) MM/GBSA energies versus docking scores obtained for the rod CNGA1 subunit. **B)** MM/GBSA energies versus docking scores obtained for the cone CNGA3 subunit. Black solid lines represent fitted linear regression curves. The Pearson correlation coefficient is indicated on the top left of the plots. **1:** 8-Br-cGMP, **2:** 8-pCPT-cGMP, **3:** PET-cGMP, **4:** Rp-cGMPS, **5:** Sp-cGMPS, **6:** Rp-8-pCPT-cGMPS, **7:** Sp-8-pCPT-cGMPS, **8:** Rp-(2-N)ET-cGMPS, **9:** Rp-1-Bn-8-Br-cGMPS, **10:** Rp-β-1-N^2-Ac-8-Br-cGMPS, **11:** Rp-8-Br-(3-Tp)ET-cGMPS, **12:** Rp-8-Br-PET-cGMPS, **13:** Rp-8-Br-αMβP-ET-cGMPS, **14:** Rp-8-Br-pMe-PET-cGMPS, **15:** Rp-8-Br-(2-N)ET-cGMPS, **16:** Rp-8-pCPT-PET-cGMPS**.**

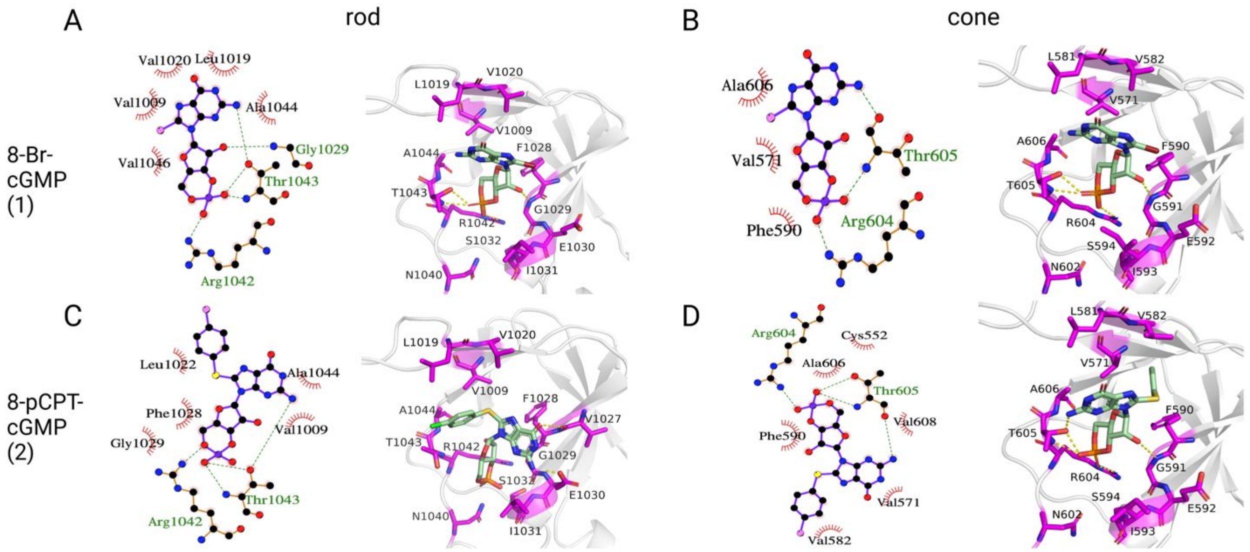

Figure S5. Binding modes of cGMP analogues with substitutions at position C8 of the guanine ring in the CNBD of CNGB-type subunit. For each ligand (**A,B**: 8-Br-cGMP; **C,D**: 8-pCPT-cGMP), 2D and 3D diagrams of their binding modes in the CNBD of (left) rod and (right) cone CNGA-type subunit are shown. Hydrophobic contacts are represented as red arcs in the 2D diagrams; hydrogen bonds are depicted as green dashed lines between amino acid residues and ligand. Residues, important for binding, as shown by their MM/GBSA energy, are colored in magenta in the 3D diagrams.

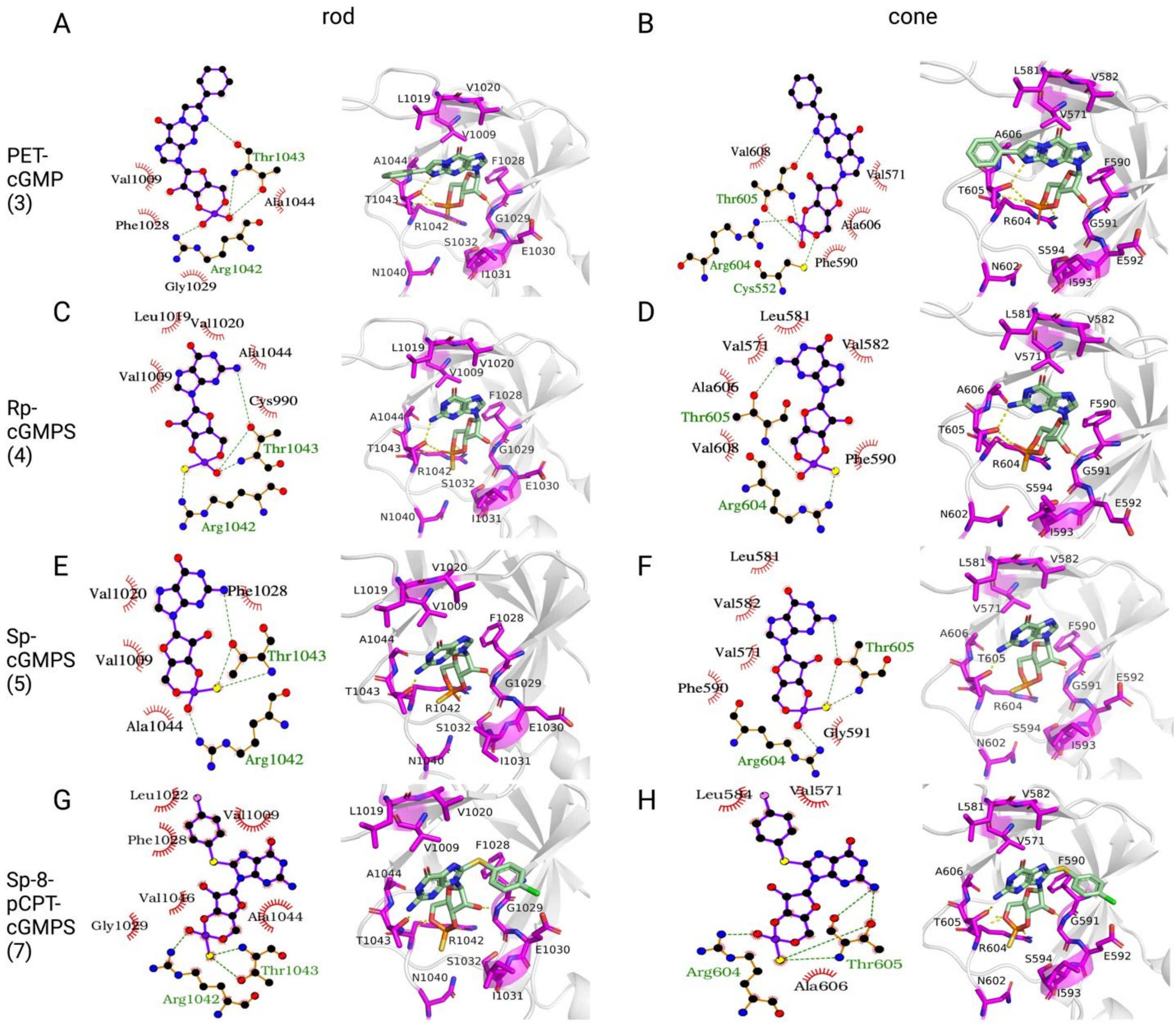

Figure S6. Binding modes of cGMP analogues with Rp-modification or substitutions at positions 1 and 2 of the nucleobase in the CNGB-type subunit. For each ligand (**A,B**: PET-cGMP; **C,D**: Rp-cGMPS; **E,F**: Sp-cGMPS; **G,H**: Sp-8-pCPT-cGMPS), 2D and 3D diagrams of their binding modes in rod (left) and cone (right) CNG channel are shown. Hydrophobic contacts are represented as red arcs in the 2D diagrams; hydrogen bonds are depicted as green dashed lines between amino acid residues and ligand. Residues, important for binding, are colored in magenta in the 3D diagrams.

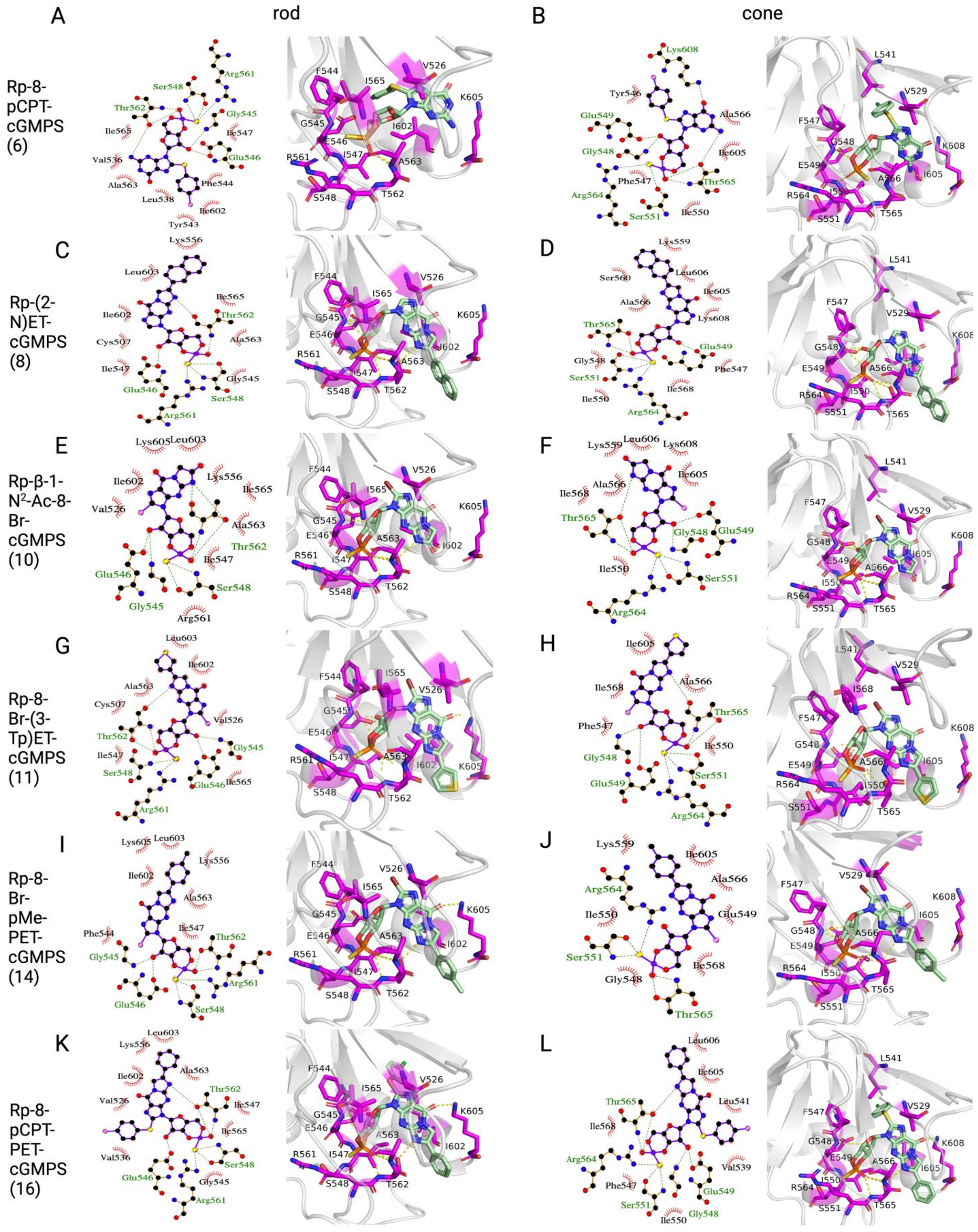

Figure S7. Binding modes of cGMP analogues with Rp-modifications at the cyclic phosphate moiety of cGMP and substitutions at N1, N^2, C8 in the CNGA-type subunit. For each ligand,

(**A,B**: Rp-8-pCPT-cGMPS; **C,D**: Rp-(2-N)ET-cGMPS; **E,F**: Rp-β-1,N^2-Ac-8-Br-cGMPS; **G,H**: Rp-8-Br-(3-Tp)ET-cGMPS; **I,J**: Rp-8-Br-pMe-PET-cGMPS; **K,L**: Rp-8-pCPT-PET-cGMPS), 2D and 3D diagrams of representative binding poses from the MD simulations are shown. Hydrophobic contacts are represented as red arcs in the 2D diagrams; hydrogen bonds are depicted as green dashed lines between amino acid residues and ligand. Residues, important for binding, are colored in magenta in the 3D diagrams.

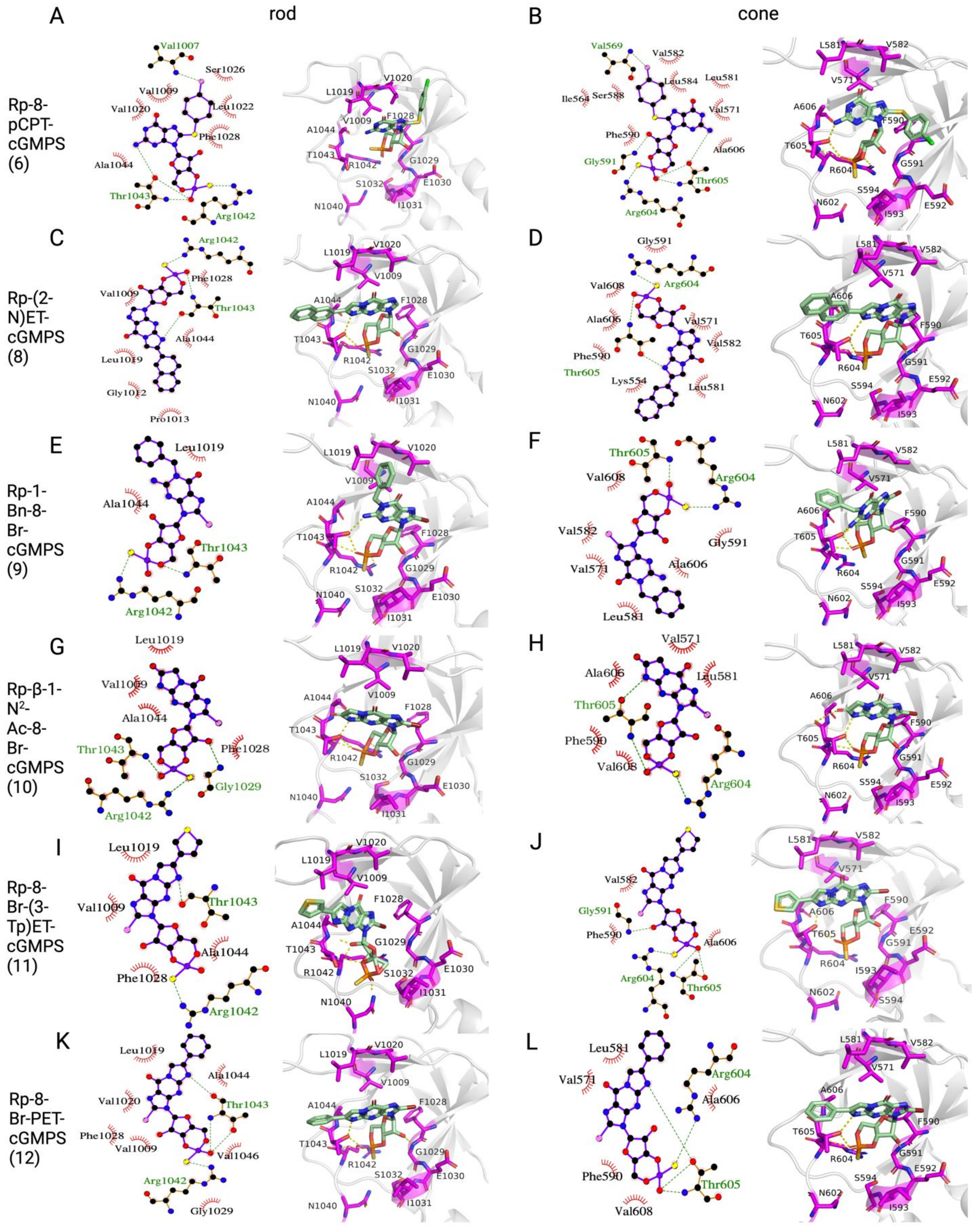
A
rod
B
cone
Rp-8-pCPT-cGMPS (6)
C
D
Rp-(2-N)ET-cGMPS (8)
E
F
Rp-1-Bn-8-Br-cGMPS (9)
G
H
Rp-β-1-N2-Ac-8-Br-cGMPS (10)
I
J
Rp-8-Br-(3-Tp)ET-cGMPS (11)
K
L
Rp-8-Br-PET-cGMPS (12)

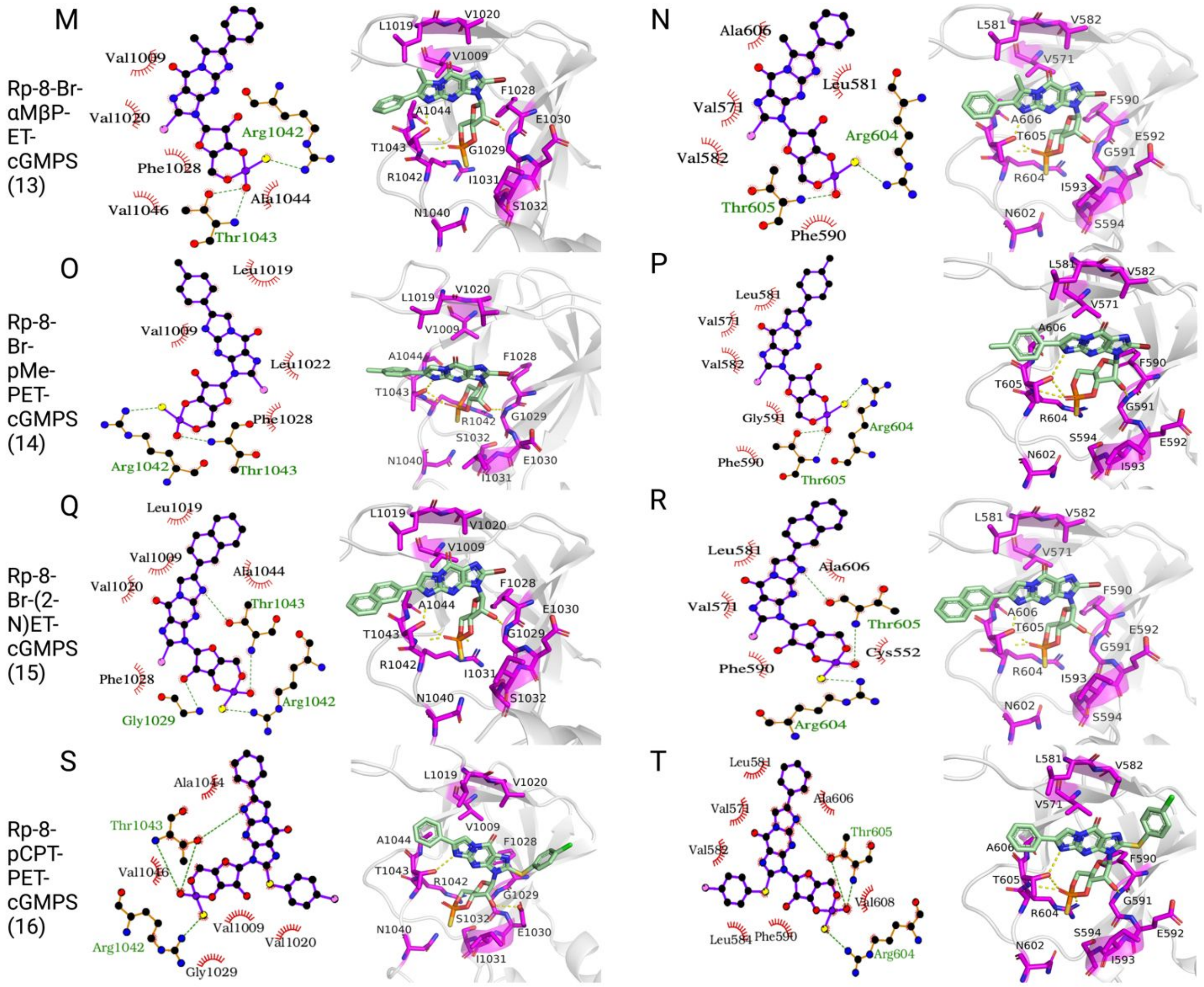

Figure S8. Binding modes of cGMP analogues with Rp-modifications at the cyclic phosphate moiety of cGMP and substitutions at N1, N^2, C8 in the CNGA-type subunit. For each ligand (**A,B**: Rp-8-pCPT-cGMPS; **C,D**: Rp-(2-N)ET-cGMPS; **E,F**: Rp-1-Bn-8-Br-cGMPS; **G,H**: Rp-β-1,N^2-Ac-8-Br-cGMPS; **I,J**: Rp-8-Br-(3-Tp)ET-cGMPS; **K,L**: Rp-8-Br-PET-cGMPS; **M,N**: Rp-8-Br-αMβP-ET-cGMPS; **O,P**: Rp-8-Br-pMe-PET-cGMPS; **Q,R**: Rp-8-Br-(2-N)ET-cGMPS; **S,T**: Rp-8-pCPT-PET-cGMPS), 2D and 3D diagrams of representative binding poses from the MD simulations are shown. Hydrophobic contacts are represented as red arcs in the 2D diagrams; hydrogen bonds are depicted as green dashed lines between amino acid residues and ligand. Residues, important for binding, are colored in magenta in the 3D diagrams.

Supplementary Tables

Supplementary Table 1. Effects of cGMP and cGMP analogues on retinal CNG channels. The table shows the EC_{50} and H (Hill coefficient) values obtained from the concentrations-activation relationships presented in Fig. 3 C,D.

Ligand (µM)	cone CNG channels			rod CNG channels		
	***EC*₅₀**	***H***	***n***	***EC*₅₀**	***H***	***n***
cGMP	18.74	1.6	6	44.95	1.67	5
8-Br-cGMP	1.04	2.0	4-7	6.18	2.0	5-11
8-pCPT-cGMP	0.09	1.7	5-11	0.77	1.6	4
Sp-cGMPS	2.88	1.2	5	-	-	-
Sp-8-pCPT-cGMPS	131.4	0.8	5	42.84	1.1	6

Supplementary Table 2. Statistical analysis of the effect of different cGMP analogues on rod and cone CNG channels. The rod CNG-channel current in the presence of the cGMP analogues at the respective concentrations was compared with the corresponding cone CNG-channel activity.

cGMP analogues	Ligand concentration	n (cone)	n (rod)	*p*-value	
Sp-cGMPS	5 mM	4	4	0.164	ns
8-Br-cGMP	1 mM	7	11	0.036	*
8-pCPT-cGMP	100 µM	5	4	0.944	ns
Sp-8-pCPT-cGMPS	1 mM	5	6	0.002	**
Rp-cGMPS	1 mM	6	8	<0.0001	****
PET-cGMP	3 mM	6	4	0.0008	***
Rp-8-pCPT-cGMPS	1 mM	7	10	0.087	ns
Rp-8-Br-(3-Tp)ET-cGMPS	1 mM	5	5	0.66	ns
Rp-8-Br-pME-PET-cGMPS	1 mM	5	4	0.773	ns
Rp-8-Br-(2-N)ET-cGMPS	1 mM	17	15	0.459	ns
Rp-(2-N)ET-cGMPS	1 mM	15	13	0.181	ns
Rp-8-Br-PET-cGMPS	1 mM	6	15	0.054	ns
Rp-8-Br-αMβP-cGMPS	1 mM	5	5	0.125	ns
Rp-β-1,N^2-Ac-8-Br-cGMPS	1 mM	12	10	0.105	ns
Rp-8-pCPT-PET-cGMPS	500 µM	4	5	0.488	ns
Rp-1-Bn-8-Br-cGMPS	1 mM	7	6	0.6833	ns
